# Inflammatory and Insulin Resistance Indicator for Predicting Repeat Revascularization and In‐Stent Restenosis in Patients With Chronic Coronary Syndrome Undergoing Percutaneous Coronary Intervention

**DOI:** 10.1002/clc.70512

**Published:** 2026-11-05

**Authors:** Wang Jiale, Liu Longcheng, Lu Peipei, Ma Lihong

**Affiliations:** ^1^ Fuwai Hospital Chinese Academy of Medical Sciences and Peking Union Medical College Beijing China

**Keywords:** C‐reactive protein‐triglyceride‐glucose index, in‐stent restenosis, percutaneous coronary intervention, repeat revascularization

## Abstract

**Objective:**

The C‐reactive protein‐triglyceride glucose index (CTI) has been associated with cardiovascular risk. This study evaluated its prognostic value for repeat revascularization and in‐stent restenosis (ISR) in patients with chronic coronary syndrome (CCS) undergoing percutaneous coronary intervention (PCI).

**Methods:**

A total of 2147 CCS patients undergoing PCI were stratified into CTI tertiles. The primary endpoint was a composite of repeat revascularization and ISR. Kaplan–Meier analysis with log‐rank tests was used to compare event rates. Multivariable Cox proportional hazards models and restricted cubic spline analyses were applied to assess associations. Predictive performance was evaluated using time‐dependent receiver operating characteristic curves.

**Results:**

During a median follow‐up of 60 months, 992 patients (46.2%) experienced the primary endpoint. Event rates increased progressively across CTI tertiles. After multivariable adjustment, CTI remained independently associated with the primary endpoint (HR 1.11; 95% CI 1.01–1.22; *p* = 0.028). Compared with the lowest tertile, the highest CTI tertile was associated with a 31% higher risk (HR 1.31; 95% CI 1.10−1.55; *p* = 0.002). A linear dose–response relationship was observed (*p* for non‐linearity = 0.172; *p* overall = 0.040). The multivariable model incorporating CTI showed modest discrimination, with AUCs of 0.627 (95% CI 0.602–0.651) at 3 years and 0.651 (95% CI 0.627–0.674) at 5 years.

**Conclusions:**

CTI is independently associated with increased risks of repeat revascularization and ISR after PCI in patients with CCS. However, its incremental predictive value beyond conventional risk factors appears limited and requires further validation.

## Graphical abstract

In patients with chronic coronary syndrome undergoing PCI, higher baseline CTI was independently associated with repeat revascularization and in‐stent restenosis. However, its incremental predictive value beyond conventional risk factors was limited, highlighting the need for further validation.

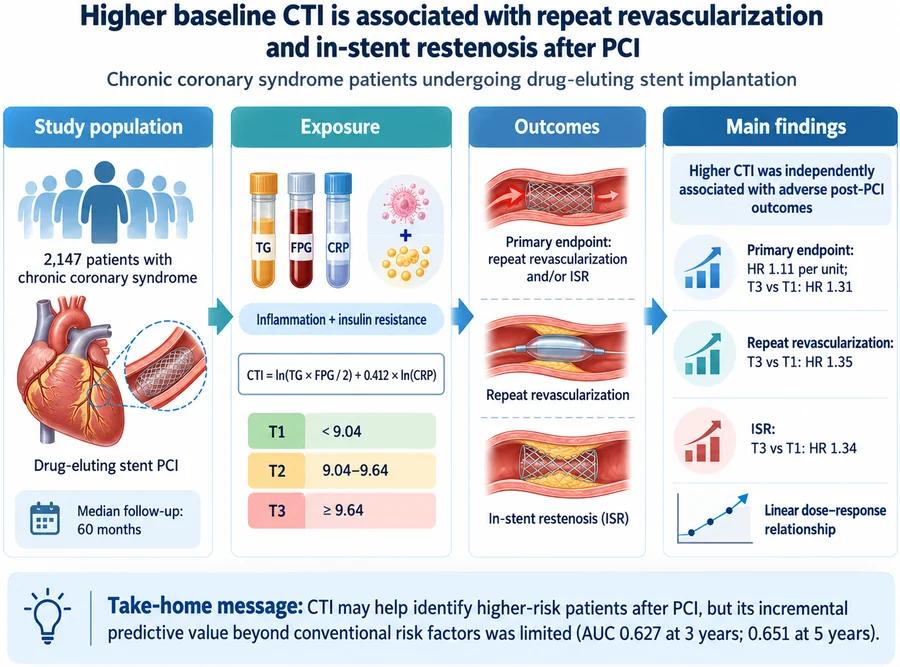

**Abbreviations**
ACEI: angiotensin‐converting enzyme inhibitor
ACS: acute coronary syndrome
AUC: area under curve
BMI: body mass index
CABG: coronary artery bypass grafting
CAD: coronary artery disease
CAG: coronary angiography
CCS: chronic coronary syndrome
CCTA: coronary CT angiography
CIs: confidence intervals
CRP: C‐reactive protein
CTI: C‐reactive protein‐triglyceride glucose index
CVD: cardiovascular disease
DAPT: dual antiplatelet therapy
DCA: decision curve analysis
DES: drug‐eluting stents
eGFR: estimated glomerular filtration rate
ESR: erythrocyte sedimentation rate
FBG: fasting blood glucose
HbA1c: glycated hemoglobin A1c
HDL‐C: high‐density lipoprotein cholesterol
HR: hazard ratio
hs‐CRP: high‐sensitive C‐reactive protein
IDI: integrated discrimination improvement
IQR: interquartile range
IR: insulin resistance
ISR: in‐stent restenosis
LDL‐C: low‐density lipoprotein cholesterol
Lp(a): lipoprotein(a)
LRT: likelihood‐ratio test
LVEF: left ventricular ejection fraction
LYMPH: lymphocytes
MI: myocardial infarction
MONO: monocytes
NEUT: neutrophils
NRI: net reclassification improvement
NT‐proBNP: N‐terminal pro‐B‐type natriuretic peptide
PAD: peripheral artery disease
PCI: percutaneous coronary intervention
Pcr: plasma creatine
RCS: restricted cubic splines
ROC: receiver operating characteristic
TC: total cholesterol
TG: triglyceride
TyG: triglyceride‐glucose
URIC: uric acid
WBC: white blood cells

## 1 Introduction

According to the 2023 data from the Global Burden of Disease Study (GBD), ischemic heart disease (IHD) remains the leading cause of global disease burden and mortality [1, 2, 3]. The term “chronic coronary syndromes (CCS)” was introduced by the 2019 ESC Guidelines to characterize stable presentations of coronary artery disease (CAD), specifically including the stabilized phase after acute coronary syndrome (ACS) or post‐revascularization [4]. In CCS patients with obstructive disease who are at high risk of adverse events and in whom guideline‐directed medical therapy fails to relieve symptoms, coronary revascularization, especially percutaneous coronary intervention (PCI) with drug‐eluting stents (DES), can improve clinical symptoms and long‐term prognosis [5].

Nevertheless, repeat revascularization and in‐stent restenosis (ISR) continue to impair long‐term outcomes and quality of life. Among patients who underwent PCI for the first time with TAXUS Express paclitaxel‐eluting stents, the incidence of repeat revascularization within 5 years was 25.9% [6], and the 10‐year mortality rate in those who experienced repeat revascularization was 33.5% [7]. Among patients using everolimus‐eluting stents, the rate of revascularization was 11% at a median follow‐up time of 4.6 years [8] and 22.6% at a median follow‐up time of 11.8 years [9]. In addition, although DES technology significantly reduces the occurrence of ISR, complications of ISR and subsequent repeat revascularization continue to arise in 1%−2% of patients each year [10]. Furthermore, treatment for ISR accounts for approximately 10% of all PCI surgeries [10]. Given that millions of DES are implanted annually around the world, identifying reliable predictors of these events is essential for refining post‐PCI risk stratification and treatment planning.

Insulin resistance (IR) is a core pathogenic engine that drives a cluster of metabolic disorders and fuels cardiovascular disease (CVD) through multiple pathways [11, 12]. The triglyceride‐glucose (TyG) index has emerged as a simple, clinically convenient tool for evaluating IR [13]. Previous studies have linked higher TyG values to worse outcomes across various CVD [14, 15, 16]. However, these previous studies have mainly concentrated on elucidating the role of IR in advancing CVD and have not explored how systemic inflammation may amplify or even mediate its effects. Inflammation is now recognized as a central accelerator of coronary disease evolution [17, 18], and accumulating evidence shows that anti‐inflammatory strategies can tangibly lower cardiovascular event rates [19, 20, 21]. C‐reactive protein (CRP), an easily measured but nonspecific inflammatory biomarker, powerfully predicts incident IHD and cardiovascular death in the general population [22], and independently forecasts new‐onset heart failure in patients with established CVD [23]. Therefore, evaluating IR in tandem with inflammation may provide a more powerful strategy for predicting and managing CVD.

Ruan et al. were the first to propose the C‐reactive protein‐triglyceride‐glucose index (CTI) [24]. By combining CRP with the TyG index, this innovative metric simultaneously gauges both IR and inflammation, thereby reflecting how chronic low‐grade inflammatory processes interrelate with metabolic dysfunction. While the CTI index has shown promise in predicting cardiovascular outcomes in various populations [25, 26, 27], its long‐term prognostic value specifically in CCS patients undergoing DES implantation remains underexplored. Therefore, we sought to evaluate the prognostic value of CTI for long‐term post‐PCI complications, including repeat revascularization and ISR, among CCS patients treated with DES implantation.

## 2 Methods

### 2.1 Study Population

This retrospective, single‐center cohort study recruited consecutive patients diagnosed with CCS who underwent PCI with DES implantation at Fuwai Hospital between March and December 2017. A total of 2147 individuals were finally included after applying the following exclusion criteria: (1) age < 18 years; (2) absence of DES implantation; (3) prior coronary artery bypass grafting (CABG); (4) failure to undergo repeat coronary angiography (CAG) or coronary CT angiography (CCTA) during follow‐up; (5) missing baseline data required for calculating the CTI index; (6) baseline fasting triglycerides (TG) ≥ 500 mg/dL raising suspicion for familial hypertriglyceridemia; (7) ISR initially identified on CCTA but not validated by CAG; (8) presence of ischemia‑driven symptoms during follow‑up but refusal of further coronary evaluation; and (9) angiographically documented disease progression at follow‑up with refusal of repeat intervention. Figure 1 summarizes how patients were selected. This study was performed in conformity with the Declaration of Helsinki and authorized by the Institutional Review Board of Fuwai Hospital. Informed consent, either written or oral, was secured from every participant.

**Figure 1:**
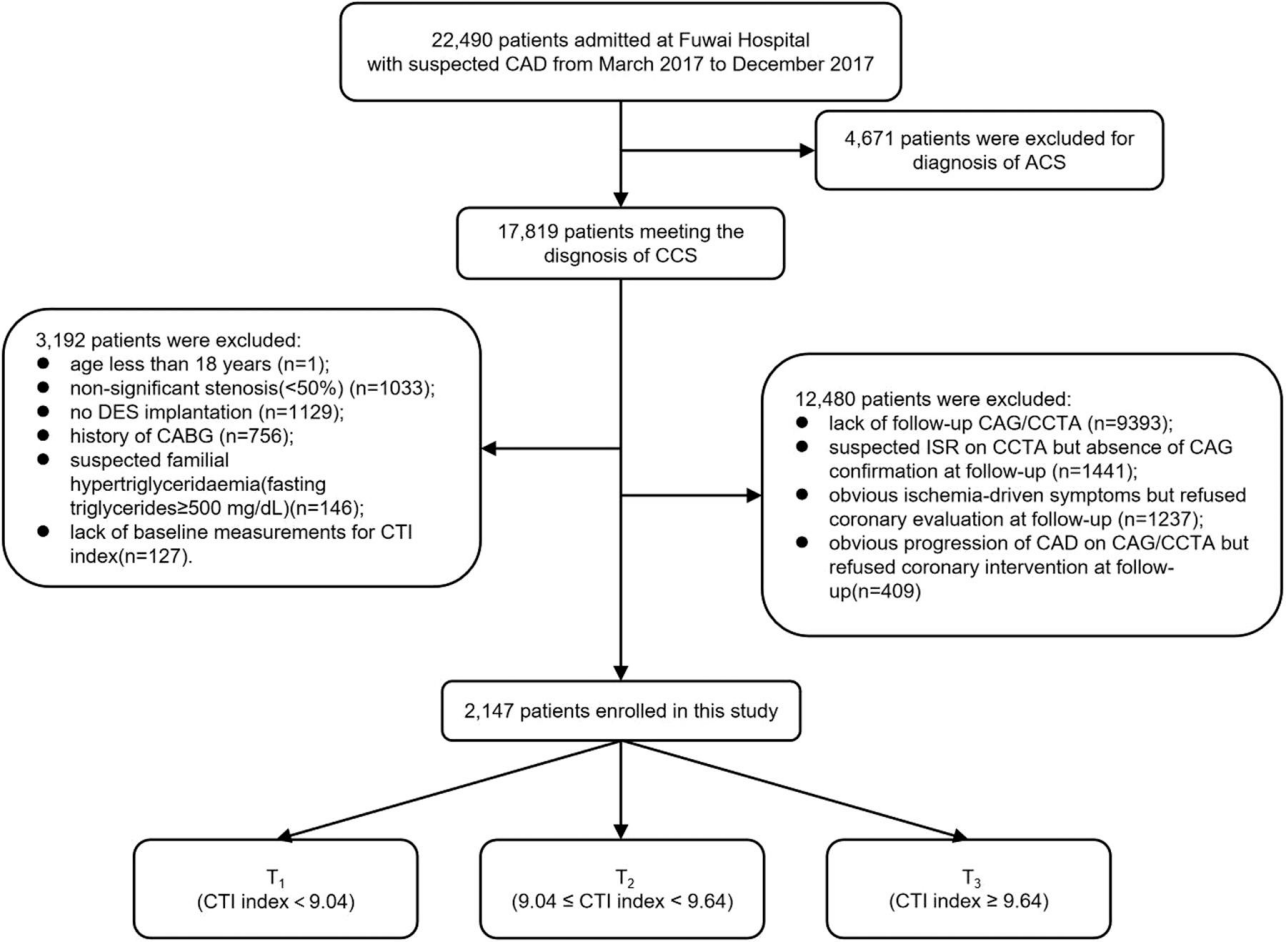
Flowchart of study participants. ACS, acute coronary syndrome; CABG, coronary artery bypass grafting; CAD, coronary artery disease; CAG, coronary angiography; CCTA, coronary CT angiography; CTI, C‐reactive protein‐triglyceride glucose index; ISR, in‐stent restenosis.

### 2.2 Data Collection and Definitions

Demographic characteristics, medical history, and discharge medications were extracted from electronic medical records. A Hitachi LABOSPECT 008 instrument (Tokyo, Japan) was employed for all assays under standardized conditions. Serum levels of CRP and hs‐CRP were measured using a high‐sensitivity immunoturbidimetric assay, while TG and other lipid parameters were determined using standard enzymatic methods. All measurements were performed in a central laboratory following standardized protocols, with reported intra‐ and inter‐assay coefficients of variation within acceptable ranges, ensuring good analytical precision and reproducibility. The comprehensive biomarker profile included inflammatory markers (CRP, hs‐CRP), metabolic indicators (FBG, TG, TC, LDL‐C, HDL‐C, URIC), renal function (Pcr), lipoprotein(a), and cardiac biomarkers (NT‐proBNP). Glycated hemoglobin (HbA1c) was quantified by high‐performance liquid chromatography (G8, TOSOH, Tokyo, Japan). All participants provided fasting samples obtained after an overnight fast before baseline CAG, ensuring standardized timing. Left ventricular ejection fraction (LVEF) was derived from biplane Simpson echocardiography. All angiographic data were extracted from institutional catheterization records.

CCS was defined in accordance with contemporary European Society of Cardiology guidelines [5]. Body mass index (BMI) was calculated as weight in kilograms divided by height in meters squared (kg/m^2^). Diabetes mellitus was diagnosed based on prior clinical diagnosis, use of glucose‑lowering agents, or meeting American Diabetes Association diagnostic criteria [28]. Based on the European Society of Hypertension recommendations, hypertension was diagnosed based on criteria including office systolic BP ≥ 140 mmHg, diastolic BP ≥ 90 mmHg, or current anti‐hypertensive treatment in the past 2 weeks [29]. Dyslipidemia was defined as abnormal plasma lipid profiles characterized by elevated levels of total cholesterol, LDL cholesterol, or TG, reduced HDL cholesterol, or any combination thereof [30]. The TyG index was calculated as Ln (fasting triglycerides [mg/dL] × fasting plasma glucose [mg/dL]/2). The value of the CTI was defined as the TyG index + 0.412 × ln (CRP [mg/L]) [24, 31]. For estimated glomerular filtration rate (eGFR), the Chinese‐adjusted Modification of Diet in Renal Disease formula was applied: 186 × [Pcr]−1.154 × [age]−0.203 × 0.742 (if female) × 1.233 [32].

### 2.3 Endpoints and Follow‐Up

The primary endpoint was a composite endpoint consisting of angiographically confirmed ISR and clinically driven repeat revascularization, while these PCI complications were separately assessed as secondary outcomes. Repeat revascularization encompassed any subsequent percutaneous or surgical intervention performed on either the initially stented vessel or other coronary segments, and included strategies such as implantation of another DES, bare‑metal stenting, conventional or drug‐eluting balloon angioplasty, and CABG. The decision to perform repeat revascularization was made by experienced interventional cardiologists according to predefined criteria, including objective evidence of ischemia (e.g., stress testing or fractional flow reserve), angiographic findings (such as ≥ 70% diameter stenosis in a major epicardial vessel or ≥ 50% in the left main artery), and clinical symptoms. Patient preference was considered after standardized physician‐patient discussions. When necessary, decisions were further reviewed by a multidisciplinary heart team to ensure consistency. To avoid over‐counting events related to staged procedures, records of planned staged PCI were excluded. ISR was defined as luminal diameter stenosis ≥ 50% within the stented segment or extending to its 5 mm margins, as confirmed by follow‐up imaging.

Follow‐up coronary evaluation was generally recommended approximately 1 year after PCI and was performed using either invasive CAG or CCTA. Study surveillance persisted until October 2022. Given the high negative predictive value of CCTA for ruling out ISR, patients without significant stenosis on CCTA were classified as event‐free. For cases with suspected ISR on noninvasive imaging, confirmatory CAG was required. Post‐procedural imaging was evaluated by expert radiologists and interventional cardiologists. All revascularization interventions and associated peri‐procedural care conformed to updated international guidelines and site‐specific protocols. All patients received the treatment recommended by the guidelines at discharge [5].

### 2.4 Statistical Analysis

Continuous variables were summarized as mean ± standard deviation for normal distributions or median and interquartile range (IQR) for skewed data. Categorical variables were summarized as frequencies and percentages (%). Participants were categorized into tertiles based on the CTI index: T_1_ (CTI < 9.04), T_2_ (9.04 ≤ CTI < 9.64), and T_3_ (CTI ≥ 9.64).

For comparisons among CTI tertiles, one‐way ANOVA or the Kruskal−Wallis test was used for continuous variables, as appropriate, and the chi‐square test was applied for categorical variables. Missing data were handled using multiple imputation via Gibbs sampling [33, 34]. Missing data were imputed using multiple imputation by chained equations (MICE). The iterative algorithm predicted missing values based on the available data set until convergence, generating five complete imputed data sets [35]. To evaluate the potential impact of missing data handling on the study results, a sensitivity analysis was performed using complete‐case data, including only patients with no missing values in any covariates included in the multivariable models.

Survival analyses were performed using Kaplan−Meier curves, and differences among tertiles were assessed with the log‐rank test. The Cox proportional hazards regression model was employed to evaluate the associations between the CTI index, TyG index, CRP, and study endpoints, respectively, with results reported as hazard ratios (HRs) and 95% confidence intervals (CIs). Multivariable models were adjusted for clinically relevant confounders, including age, gender, BMI, diabetes, hypertension, previous PCI, previous myocardial infarction (MI), HbA1c, Lp(a), HDL‐C, presence of multivessel CAD, presence of lesion length ≥ 20 mm, number of stents, and stent length. Restricted cubic splines (RCS) with three knots (placed at the 10th, 50th, and 90th percentiles of the CTI distribution) were used to examine potential nonlinear relationships between the CTI index and endpoints [34, 36]. The CTI median functioned as the reference standard. In addition, the predictive performance of the models was evaluated using receiver operating characteristic (ROC) curves, with the area under the curve (AUC) metric quantifying the model's discriminative ability. Model discrimination was further assessed using Harrell's concordance index (C‐index). To evaluate the incremental prognostic value of CTI beyond conventional risk factors, the baseline model and the CTI‐enhanced model were compared using likelihood‐ratio testing (LRT), net reclassification improvement (NRI), and integrated discrimination improvement (IDI). Time‐dependent ROC analyses were performed at 3 and 5 years. Decision‐curve analysis (DCA) was performed to quantify the clinical net benefit of the CTI index. Because only a single data set was available and no external cohort was accessible for validation, internal validation was performed by Bootstrap resampling. Calibration plots were used to assess model agreement: the *x*‐axis shows the model‐predicted probabilities and the *y*‐axis shows the observed event probabilities. The reference line is the 45°line of identity passing through the origin, and the closer the calibration curve lies to this line, the better the agreement between prediction and observation. All statistical analyses were performed in R software (version 4.1.2).

## 3 Results

### 3.1 Baseline Characteristics

The median age of the 2147 CCS participants undergoing DES‐PCI was 59.00 (52.00, 65.00) years, and 1661 (77.36%) were male. Higher BMI was observed in T_2_ and T_3_ groups compared with T_1_ (*p* < 0.001). Cardiometabolic risk factors (diabetes, hypertension, dyslipidemia, previous stroke) and corresponding pharmacotherapy (β‐blockers, insulin, oral hypoglycemics) were more prevalent in T_3_ group versus T_1_ and T_2_ (all *p* < 0.05). The levels of inflammation‐related markers, including WBC, NEUT, LYMPH, MONO, ESR, CRP, and hs‐CRP, showed an upward trend from T_1_ through T_3_ (all *p* < 0.05). Notably, T_3_ subjects displayed higher concentrations of lipids (TC, LDL‐C, TG), metabolic markers (FPG, HbA1c), and renal parameters (URIC, Pcr), while HDL‐C and Lp(a) levels were lower compared with those in the lower tertiles (all *p* < 0.001) (Table 1).

**Table 1:** Baseline characteristics according to tertiles of the CTI.

Variable	Total (*n* = 2147)	T_1_ (*n* = 716)	T_2_ (*n* = 715)	T_3_ (*n* = 716)	*p* value
Demographics					
Age (years)	59.00 (52.00, 65.00)	60.00 (53.75, 66.00)	59.00 (52.00, 65.00)	58.00 (51.00, 64.00)	< 0.001
Male sex, *n* (%)	1661 (77.36)	566 (79.05)	536 (74.97)	559 (78.07)	0.156
BMI (kg/m^2^)	25.95 (24.00, 28.03)	25.05 (23.15, 27.22)	25.99 (24.05, 28.12)	26.73 (24.60, 28.73)	< 0.001
Risk factors					
Cigarette smoking, *n* (%)	1293 (60.22)	431 (60.20)	413 (57.76)	449 (62.71)	0.161
Diabetes, *n* (%)	821 (38.24)	183 (25.56)	253 (35.38)	385 (53.77)	< 0.001
Hypertension, *n* (%)	1404 (65.39)	434 (60.61)	477 (66.71)	493 (68.85)	0.003
Dyslipidemia, *n* (%)	1907 (88.82)	614 (85.75)	636 (88.95)	657 (91.76)	0.001
Previous MI, *n* (%)	353 (16.44)	124 (17.32)	117 (16.36)	112 (15.64)	0.692
Previous stroke, *n* (%)	238 (11.09)	56 (7.82)	87 (12.17)	95 (13.27)	0.002
Previous PCI, *n* (%)	621 (28.92)	222 (31.01)	200 (27.97)	199 (27.79)	0.321
PAD, *n* (%)	266 (12.39)	101 (14.11)	76 (10.63)	89 (12.43)	0.136
Family history of CAD, *n* (%)	404 (18.82)	122 (17.04)	136 (19.02)	146 (20.39)	0.264
Clinical presentations					
Multi‐vessel CAD, *n* (%)	1733 (80.72)	564 (78.77)	575 (80.42)	594 (82.96)	0.129
LVEF (%)	63.00 (60.00, 65.00)	63.00 (60.00, 66.00)	63.00 (60.00, 65.00)	63.00 (60.00, 65.00)	0.039
Laboratory measurements					
WBC (10^9^/L)	6.54 (5.54, 7.75)	6.08 (5.19, 7.04)	6.64 (5.62, 7.83)	7.03 (5.94, 8.35)	< 0.001
NEUT (10^9^/L)	4.08 (3.30, 5.07)	3.71 (3.01, 4.49)	4.20 (3.38, 5.11)	4.43 (3.56, 5.52)	< 0.001
LYMPH (10^9^/L)	1.78 (1.45, 2.21)	1.68 (1.39, 2.06)	1.78 (1.44, 2.21)	1.89 (1.53, 2.33)	< 0.001
MONO (10^9^/L)	0.39 (0.31, 0.50)	0.36 (0.29, 0.46)	0.38 (0.31, 0.49)	0.42 (0.33, 0.54)	< 0.001
TC (mmol/L)	3.95 (3.35, 4.71)	3.62 (3.09, 4.25)	3.94 (3.39, 4.62)	4.35 (3.65, 5.14)	< 0.001
LDL‐C (mmol/L)	2.31 (1.82, 2.94)	2.08 (1.66, 2.64)	2.36 (1.88, 2.92)	2.50 (1.99, 3.19)	< 0.001
HDL‐C (mmol/L)	1.06 (0.90, 1.26)	1.16 (0.99, 1.38)	1.06 (0.90, 1.26)	0.98 (0.84, 1.14)	< 0.001
Triglycerides (mmol/L)	1.46 (1.10, 2.02)	1.02 (0.82, 1.23)	1.54 (1.30, 1.86)	2.25 (1.69, 3.04)	< 0.001
FPG (mmol/L)	5.67 (5.05, 6.92)	5.24 (4.80, 5.77)	5.61 (5.05, 6.62)	6.79 (5.61, 8.99)	< 0.001
HbA1c (%)	6.00 (5.60, 6.80)	5.75 (5.50, 6.20)	5.90 (5.60, 6.70)	6.60 (5.88, 7.70)	< 0.001
ESR (mm/h)	7.00 (3.00, 13.00)	6.00 (2.00, 10.00)	7.00 (3.00, 13.00)	10.00 (5.00, 17.00)	< 0.001
URIC (μmol/L)	346.80 (290.00, 401.31)	332.47 (280.87, 389.64)	343.35 (285.11, 403.49)	360.71 (302.50, 412.53)	< 0.001
Lp(a) (nmol/L)	159.66 (69.40, 413.95)	168.57 (76.42, 460.78)	177.90 (76.80, 418.26)	138.22 (59.79, 362.69)	< 0.001
Pcr (μmol/L)	78.81 (69.26, 89.46)	78.05 (69.02, 87.95)	78.81 (68.83, 89.00)	79.81 (70.00, 91.16)	0.040
eGFR (mL/min per 1.73 m^2^)	108.84 (94.62, 123.45)	109.95 (96.49, 123.84)	108.29 (95.97, 123.00)	108.24 (91.80, 123.29)	0.106
NT‐proBNP (pg/mL)	84.20 (37.55, 192.35)	78.50 (39.32, 186.98)	82.40 (34.40, 179.65)	88.60 (36.80, 203.20)	0.616
CRP (mg/L)	2.71 (1.85, 4.56)	1.93 (1.46, 2.52)	2.73 (1.94, 4.00)	4.37 (2.91, 8.10)	< 0.001
hs‐CRP (mg/L)	1.58 (0.83, 3.24)	0.90 (0.52, 1.52)	1.58 (0.90, 2.77)	2.96 (1.61, 6.80)	< 0.001
Medications at discharge					
DAPT, *n* (%)	2141 (99.72)	714 (99.72)	713 (99.72)	714 (99.72)	1.000
Statins, *n* (%)	2115 (98.51)	708 (98.88)	707 (98.88)	700 (97.77)	0.132
ACEIs, *n* (%)	1145 (53.33)	356 (49.72)	390 (54.55)	399 (55.73)	0.054
β‐Blockers, *n* (%)	1832 (85.33)	596 (83.24)	606 (84.76)	630 (87.99)	0.035
Insulin, *n* (%)	241 (11.22)	65 (9.08)	58 (8.11)	118 (16.48)	< 0.001
Oral hypoglycemic drugs, *n* (%)	555 (25.85)	159 (22.21)	164 (22.94)	232 (32.40)	< 0.001
Angiographic findings					
Restenotic lesions, *n* (%)	84 (3.91)	26 (3.63)	25 (3.50)	33 (4.61)	0.496
Chronic total occlusions, *n* (%)	343 (15.98)	97 (13.55)	120 (16.78)	126 (17.60)	0.086
Lesions > 20 mm long, *n* (%)	1667 (77.64)	531 (74.16)	562 (78.60)	574 (80.17)	0.018
Number of stents	2.00 (1.00, 2.00)	2.00 (1.00, 2.00)	2.00 (1.00, 2.00)	2.00 (1.00, 2.00)	0.364
Length of stent (mm)	35.00 (20.00, 55.50)	33.00 (19.00, 55.00)	35.00 (21.00, 55.00)	35.00 (21.00, 56.00)	0.268

Abbreviations: ACEI, angiotensin‐converting enzyme inhibitor; BMI, body mass index; CAD, coronary artery disease; CRP, C‐reactive protein; DAPT, dual antiplatelet therapy; eGFR, estimated glomerular filtration rate; ESR, erythrocyte sedimentation rate; FPG, fasting plasma glucose; HbA1c, glycated hemoglobin A1c; HDL‐C, high‐density lipoprotein‐cholesterol; hs‐CRP, hypersensitive C‐reactive protein; LDL‐C, low‐density lipoprotein‐cholesterol; Lp(a), lipoprotein(a); LVEF, left ventricular ejection fraction; LYMPH, lymphocytes; MI, myocardial infarction; MONO, monocytes; NEUT, neutrophils; NT‐proBNP, N‐terminal pro‐B‐type natriuretic peptide; PAD, peripheral arterial disease; PCI, percutaneous coronary intervention; Pcr, plasma creatine; TC, total cholesterol; URIC, uric acid; WBC, white blood cells.

Table 2 summarizes baseline characteristics by primary endpoint status. During follow‐up, 992 participants developed repeat revascularization or ISR. Compared with event‐free individuals, those experiencing endpoint events exhibited higher BMI and greater prevalence of diabetes, hypertension, prior MI, prior PCI, multi‐vessel CAD, and insulin or oral hypoglycemic use(all *p* < 0.05). Additionally, event cases exhibited elevated WBC, NEUT, TG, FPG, HbA1c, Lp(a), and reduced HDL‐C (all *p* < 0.05). Endpoint cases also demonstrated higher CTI levels (*p* < 0.05), along with increased prevalence of restenotic lesions and chronic total occlusions (CTO), as well as greater stent count, total stented length, and the prevalence of lesions > 20 mm in length (all *p* < 0.05). Secondary endpoint data are provided in Supporting Information S1: Tables S1 and S2.

**Table 2:** Baseline characteristics according to the primary endpoint.

Variable	No such events (*n* = 1155)	Primary endpoint (*n* = 992)	*p* value
Demographics			
Age (years)	59.00 (52.00, 65.00)	58.00 (52.00, 65.00)	0.109
Male sex, *n* (%)	878 (76.02)	783 (78.93)	0.108
BMI (kg/m^2^)	25.71 (23.80, 27.78)	26.03 (24.22, 28.21)	0.006
Risk factors			
Cigarette smoking, *n* (%)	693 (60.00)	600 (60.48)	0.819
Diabetes, *n* (%)	389 (33.68)	432 (43.55)	< 0.001
Hypertension, *n* (%)	727 (62.94)	677 (68.25)	0.01
Dyslipidemia, *n* (%)	1018 (88.14)	889 (89.62)	0.278
Previous MI, *n* (%)	152 (13.16)	201 (20.26)	< 0.001
Previous stroke, *n* (%)	120 (10.39)	118 (11.90)	0.268
Previous PCI, *n* (%)	257 (22.25)	364 (36.69)	< 0.001
PAD, *n* (%)	152 (13.16)	114 (11.49)	0.242
Family history of CAD, *n* (%)	210 (18.18)	194 (19.56)	0.417
Clinical presentations			
Multi‐vessel CAD, *n* (%)	864 (74.81)	869 (87.60)	< 0.001
LVEF (%)	63.00 (60.00, 65.00)	63.00 (60.00, 65.00)	0.107
Laboratory measurements			
WBC (10^9^/L)	6.39 (5.43, 7.71)	6.64 (5.67, 7.80)	0.002
NEUT (10^9^/L)	3.98 (3.22, 4.95)	4.21 (3.41, 5.20)	< 0.001
LYMPH (10^9^/L)	1.78 (1.43, 2.20)	1.78 (1.45, 2.22)	0.579
MONO (10^9^/L)	0.38 (0.30, 0.49)	0.39 (0.32, 0.50)	0.225
TC (mmol/L)	3.95 (3.34, 4.64)	3.95 (3.37, 4.81)	0.168
LDL‐C (mmol/L)	2.29 (1.79, 2.88)	2.33 (1.84, 3.00)	0.078
HDL‐C (mmol/L)	1.09 (0.91, 1.28)	1.03 (0.88, 1.24)	< 0.001
Triglycerides (mmol/L)	1.43 (1.07, 1.96)	1.51 (1.14, 2.13)	0.002
FPG (mmol/L)	5.51 (5.00, 6.59)	5.91 (5.12, 7.35)	< 0.001
HbA1c (%)	5.90 (5.60, 6.60)	6.10 (5.70, 7.10)	< 0.001
ESR (mm/h)	7.00 (3.00, 13.00)	7.00 (3.00, 14.00)	0.437
URIC (μmol/L)	347.56 (291.09, 402.57)	346.40 (288.55, 399.54)	0.481
Lp(a) (nmol/L)	148.16 (66.20, 391.50)	174.25 (75.95, 451.30)	0.005
Pcr (μmol/L)	78.67 (68.97, 88.99)	79.16 (69.58, 90.02)	0.203
eGFR (mL/min per 1.73 m^2^)	108.40 (95.44, 121.91)	109.31 (93.49, 125.11)	0.753
NT‐proBNP (pg/mL)	81.20 (33.80, 177.75)	86.35 (40.10, 203.00)	0.051
CRP (mg/L)	2.62 (1.82, 4.52)	2.82 (1.91, 4.58)	0.206
hs‐CRP (mg/L)	1.51 (0.79, 3.19)	1.66 (0.86, 3.36)	0.057
Medications at discharge			
DAPT, *n* (%)	1151 (99.65)	990 (99.80)	0.823
Statins, *n* (%)	1139 (98.61)	976 (98.39)	0.664
ACEIs, *n* (%)	612 (52.99)	533 (53.73)	0.731
β‐Blockers, *n* (%)	979 (84.76)	853 (85.99)	0.423
Insulin, *n* (%)	101 (8.74)	140 (14.11)	< 0.001
Oral hypoglycemic drugs, *n* (%)	272 (23.55)	283 (28.53)	0.009
Angiographic findings			
Restenotic lesions, *n* (%)	29 (2.51)	55 (5.54)	< 0.001
Chronic total occlusions, *n* (%)	160 (13.85)	183 (18.45)	0.004
Lesions > 20 mm long, *n* (%)	855 (74.03)	812 (81.85)	< 0.001
Number of stents	2.00 (1.00, 2.00)	2.00 (1.00, 3.00)	< 0.001
Length of stent (mm)	33.00 (19.00, 52.00)	36.00 (22.75, 58.00)	< 0.001
CTI index	9.24 (8.84, 9.74)	9.41 (8.96, 9.88)	< 0.001
CTI tertiles			< 0.001
T_1_, *n* (%)	438 (61.17)	278 (38.83)	
T_2_, *n* (%)	375 (52.45)	340 (47.55)	
T_3_, *n* (%)	342 (47.77)	374 (52.23)	

Abbreviations: ACEI, angiotensin‐converting enzyme inhibitor; BMI, body mass index; CAD, coronary artery disease; CRP, C‐reactive protein; CTI, C‐reactive protein‐triglyceride glucose index; DAPT, dual antiplatelet therapy; eGFR, estimated glomerular filtration rate; ESR, erythrocyte sedimentation rate; FPG, fasting plasma glucose; HbA1c, glycated hemoglobin A1c; HDL‐C, high‐density lipoprotein‐cholesterol; hs‐CRP, hypersensitive C‐reactive protein; LDL‐C, low‐density lipoprotein‐cholesterol; Lp(a), lipoprotein(a); LVEF, left ventricular ejection fraction; LYMPH, lymphocytes; MI, myocardial infarction; MONO, monocytes; NEUT, neutrophils; NT‐proBNP, N‐terminal pro‐B‐type natriuretic peptide; PAD, peripheral arterial disease; PCI, percutaneous coronary intervention; Pcr, plasma creatine; T, tertile; TC, total cholesterol; URIC, uric acid; WBC, white blood cells.

### 3.2 Association Between the CTI Index and the Primary Endpoint

The primary endpoint occurred in 992 individuals (46.20%) during a median follow‐up period of 60 months (IQR 16−68 months). The incidence of the primary endpoint increased across CTI tertiles, with event rates of 38.8%, 47.6%, and 52.2% in the T_1_, T_2_, and T_3_, respectively. This corresponded to an absolute risk difference of 13.4% between the highest and lowest tertiles. A graded increase in absolute risk was observed across tertiles. As illustrated in Figure 2, Kaplan−Meier analyses revealed that T_3_ exhibited markedly greater event incidence relative to T_1_ (log‐rank test, overall *p* < 0.001), attributable to elevated rates of both repeat revascularization (log‐rank test, overall < 0.001) and ISR (log‐rank test, overall *p* = 0.003).

**Figure 2:**
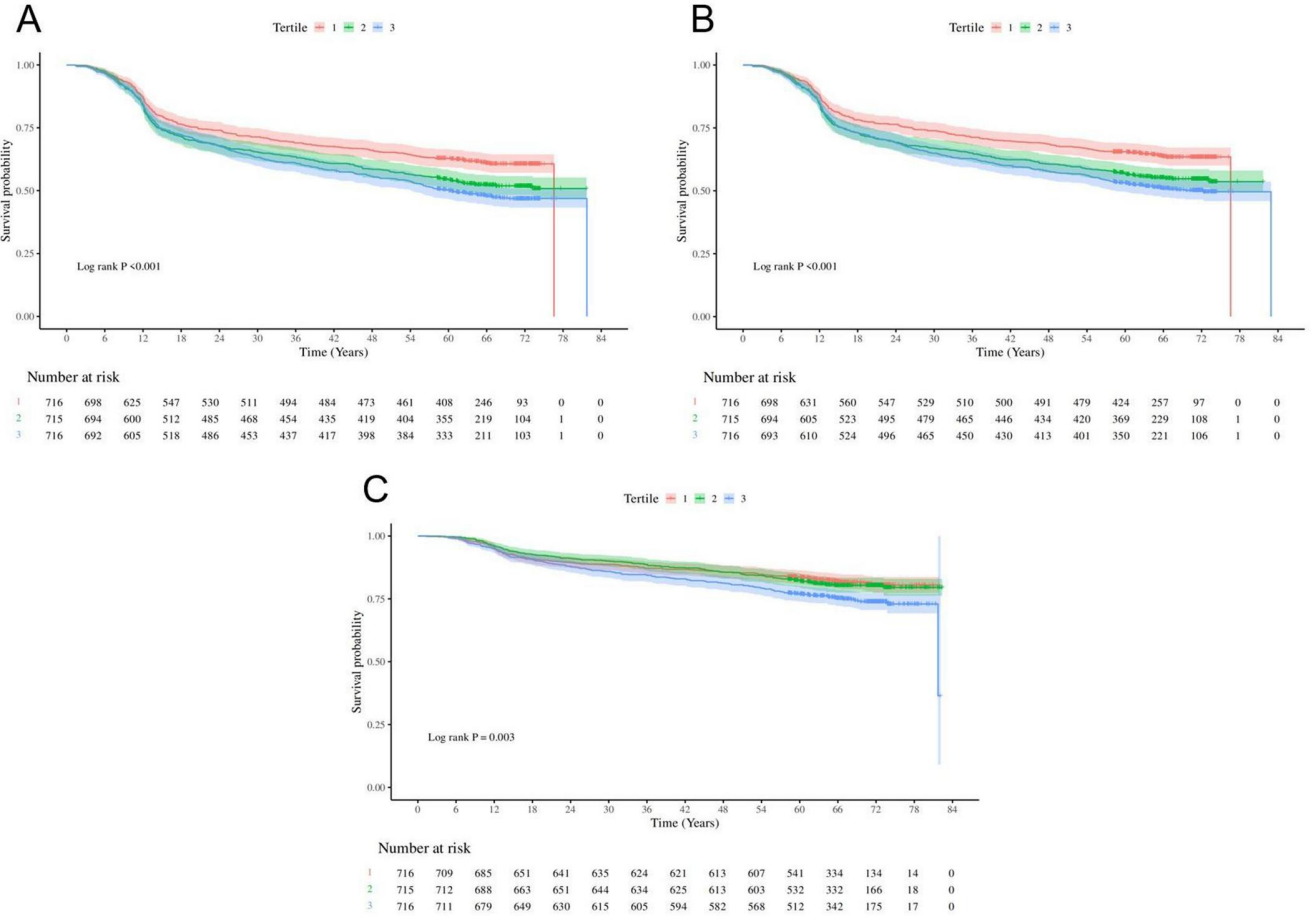
Kaplan−Meier survival analyses for the incidences of the primary endpoint and secondary endpoints among the CTI tertiles. (A) The primary endpoint including repeat revascularization and/or ISR; (B) repeat revascularization; and (C) ISR. CTI, C‐reactive protein‐triglyceride glucose index; ISR, in‐stent restenosis.

The results of Cox regression analyses are summarized in Table 3. In the unadjusted model 1, CTI significantly predicted the primary endpoint (HR, 1.20; 95% CI 1.10−1.30; *p* < 0.001), with T_3_ participants exhibiting heightened risk (HR, 1.44; 95% CI 1.24−1.69; *p* < 0.001). After adjusting for age, gender, BMI, diabetes, hypertension, previous PCI, previous MI, HbA1c, Lp(a), and HDL‐C in model 2, continuous CTI independently predicted the primary endpoint (HR, 1.13; 95% CI 1.03−1.24; *p* = 0.011). T_3_ participants faced a 33% higher risk than T_1_ (HR, 1.33; 95% CI 1.12−1.57; *p* = 0.001). Additional adjustment for anatomical complexity and stent parameters (model 3) attenuated but preserved CTI's independent association (HR, 1.11; 95% CI 1.01−1.22; *p* = 0.028). T_3_ remained at 31% higher risk versus T_1_ (HR, 1.31; 95% CI 1.10−1.55; *p* = 0.002). Sensitivity analyses further supported the robustness of our findings. First, a complete‐case analysis excluding one patient with missing lipoprotein(a) yielded results nearly identical to those of the primary analysis based on multiple imputation, with no material changes in effect estimates or statistical significance (Supporting Information S1: Table S3). Second, to minimize potential confounding from pre‐existing high‐risk coronary conditions, we performed an additional sensitivity analysis after excluding patients with prior MI, prior PCI, restenotic lesions, and CTO lesions. The associations between CTI and the primary endpoint, repeat revascularization, and ISR remained largely consistent in the fully adjusted models (Supporting Information S1: Table S4).

**Table 3:** Associations between the CTI index and the primary endpoint.

	Model 1			Model 2			Model 3		
	HR	95% CI	*p* value	HR	95% CI	*p* value	HR	95% CI	*p* value
CTI index	1.20	1.10−1.30	< 0.001	1.13	1.03−1.24	0.011	1.11	1.01−1.22	0.028
CTI tertiles									
T_1_	Reference			Reference			Reference		
T_2_	1.30	1.11−1.53	0.001	1.26	1.07−1.48	0.006	1.25	1.06−1.47	0.007
T_3_	1.44	1.24−1.69	< 0.001	1.33	1.12−1.57	0.001	1.31	1.10−1.55	0.002
TyG index	1.27	1.15−1.40	< 0.001	1.18	1.05−1.31	0.004	1.16	1.04−1.30	0.008
TyG tertiles									
T_1_	Reference			Reference			Reference		
T_2_	1.19	1.01−1.39	0.035	1.13	0.96−1.33	0.138	1.14	0.97−1.33	0.123
T_3_	1.37	1.18−1.60	< 0.001	1.23	1.04−1.46	0.017	1.20	1.02−1.43	0.031
CRP	1.00	0.99−1.00	0.421	1.00	0.99−1.00	0.418	1.00	0.99−1.00	0.279
CRP tertiles									
T_1_	Reference			Reference			Reference		
T_2_	1.01	0.87−1.18	0.880	0.99	0.85−1.16	0.893	0.97	0.83−1.13	0.715
T_3_	1.05	0.90−1.22	0.527	1.03	0.88−1.20	0.728	1.00	0.86−1.17	0.951

*Note:* Model 1: Unadjusted. Model 2: Adjusted for age, gender, BMI, diabetes, hypertension, previous PCI, previous MI, HbA1c, Lp(a), HDL‐C. Model 3: Adjusted for age, gender, BMI, diabetes, hypertension, previous PCI, previous MI, HbA1c, Lp(a), HDL‐C, presence of multivessel CAD, presence of lesion's length ≥ 20 mm, number of stents, stent length.

Abbreviations: BMI, body mass index; CAD, coronary artery disease; CI, confidence interval; CRP, C‐reactive protein; CTI, C‐reactive protein‐triglyceride glucose index; HbA1c, glycated hemoglobin A1c; HDL‐C, high‐density lipoprotein‐cholesterol; HR, hazard ratio; Lp(a), lipoprotein(a); MI, myocardial infarction; PCI, percutaneous coronary intervention; TyG, triglyceride‐glucose index.

As a continuous variable, in the fully adjusted model, both CTI (HR 1.11, 95% CI 1.01−1.22; *p* = 0.028) and TyG (HR 1.16, 95% CI 1.04−1.30; *p* = 0.008) remained significantly associated with the primary endpoint. When analyzed by tertiles, the highest CTI tertile showed an HR of 1.31 (95% CI 1.10−1.55), compared with 1.20 (95% CI 1.02−1.43) for the highest TyG tertile. In contrast, CRP was not significantly associated with the primary endpoint in any model (Table 3). These findings indicate significant associations for both CTI and TyG, without establishing the superiority of either index in predictive performance (Table 3).

Figure 3A illustrates the CTI‐primary endpoint association in Model 3 using RCS. A linear dose−response relationship was evident (nonlinear *p* = 0.172, overall *p* = 0.040).

**Figure 3:**
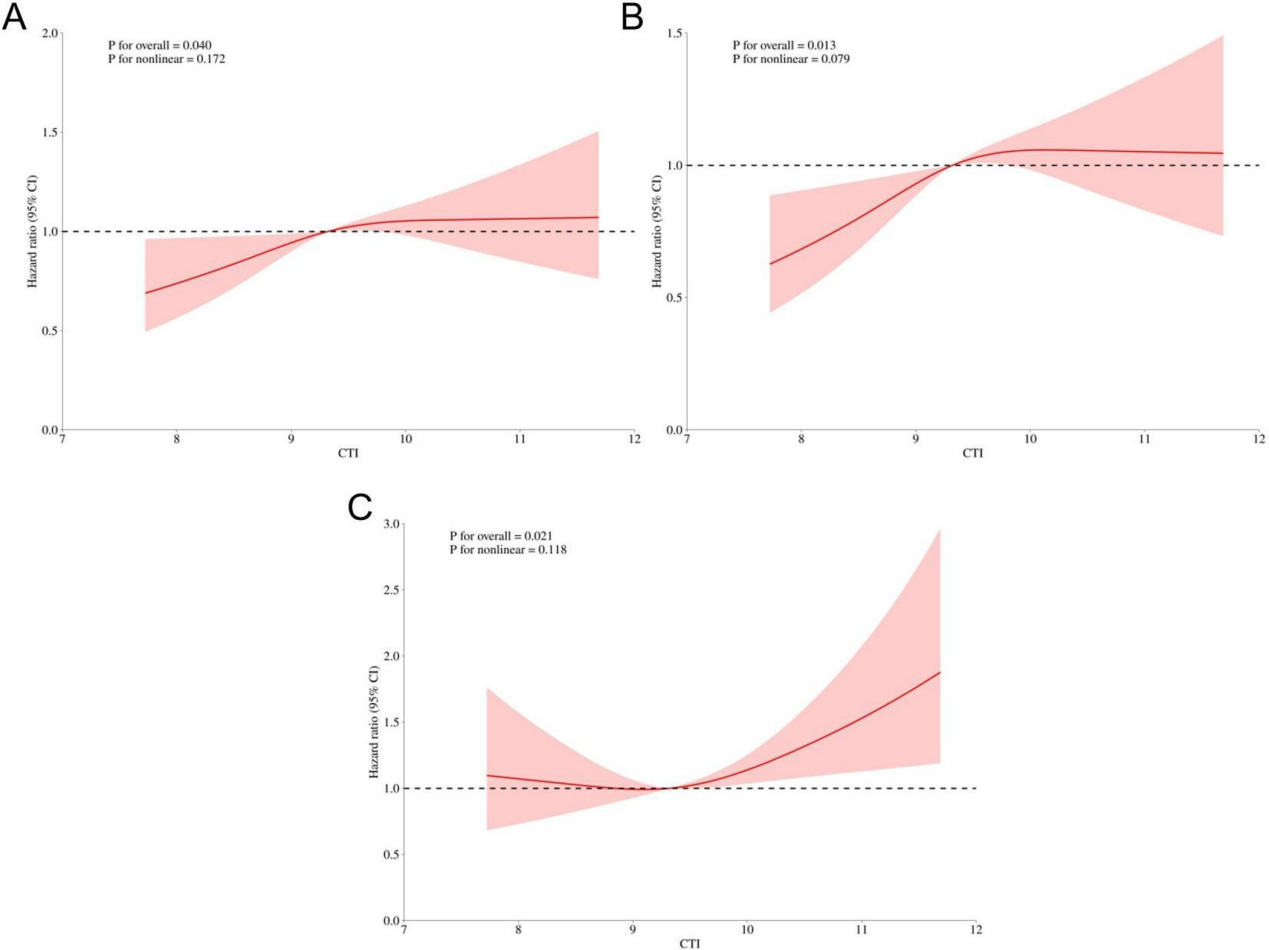
Restricted cubic splines for the adjusted dose−response association of the CTI index for the primary and secondary endpoints. Restricted cubic spline analyses were performed within multivariable Cox proportional hazards models, using three knots at the 10th, 50th, and 90th percentiles of the CTI distribution. *Y*‐axis represents the hazard ratio, and the dashed lines are 95% confidence intervals. (A) The primary endpoint including repeat revascularization and/or ISR; (B) repeat revascularization; and (C) ISR. CI, confidence interval; CTI, C‐reactive protein‐triglyceride glucose index; HR, hazard ratio; ISR, in‐stent restenosis.

The prognostic model included the CTI index, age, gender, BMI, diabetes, hypertension, previous PCI, previous MI, HbA1c, lipoprotein(a), HDL‐C, presence of multivessel CAD, lesion length ≥ 20 mm, number of stents, and total stent length. ROC analysis showed that the model incorporating the CTI index demonstrated modest discriminative ability for the primary endpoint, with AUC values of 0.627 (95% CI 0.602−0.651) at 3 years and 0.651 (95% CI 0.627−0.674) at 5 years (Figure 4A,D). Compared with the baseline clinical model, the addition of CTI resulted in numerically higher AUCs and *C*‐statistics. However, the improvements in risk reclassification and discrimination were modest and did not reach statistical significance, with an NRI of 0.054 (95% CI −0.021 to 0.095, *p* = 0.160), an IDI of 0.0014 (95% CI −0.0001 to 0.0053, *p* = 0.093), and a likelihood‐ratio test statistic of 3.247 (*p* = 0.072) (Supporting Information S1: Table S5). Nevertheless, DCA demonstrated that the model incorporating CTI provided a higher net benefit than both the “treat‐all” and “treat‐none” strategies across a range of threshold probabilities, supporting its potential clinical utility (Figure 4B,E). In addition, internal validation using 1000 bootstrap resamples demonstrated good calibration of the model, with close agreement between predicted and observed risks at both 3 and 5 years (Figure 4C,F).

**Figure 4:**
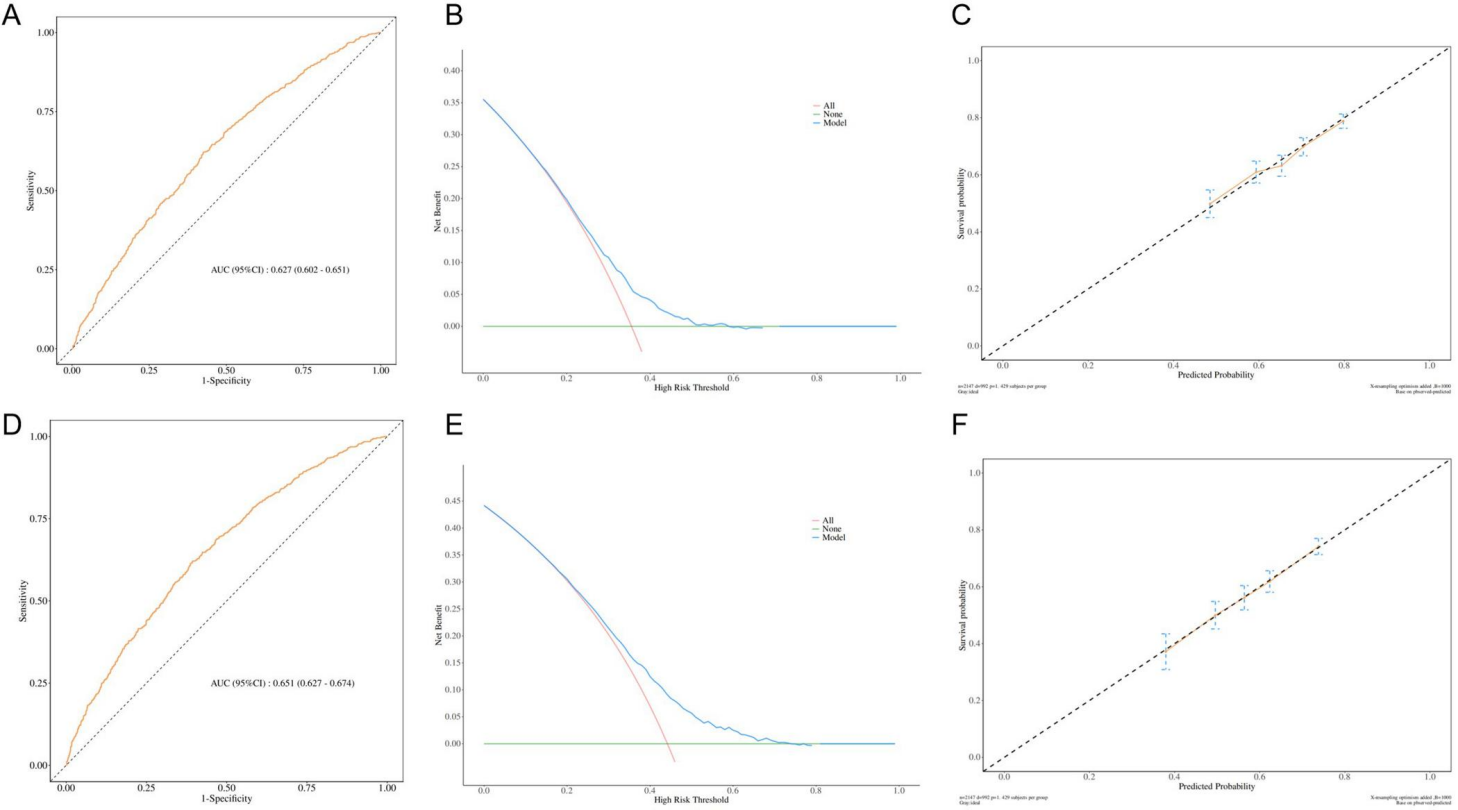
The predictive ability of CTI index for the primary endpoint was determined via the ROC analysis (A, D), decision curve analysis (DCA) (B, E), and calibration plot (C, F). (A−C) Display the 3‐year models; (D−F) show the corresponding 5‐year analyses.

To assess the stability of the association between CTI and the primary endpoint, subgroup analyses were performed, and interaction tests were conducted (Table 4). Overall, the direction of the association between CTI and the primary endpoint was generally consistent across most subgroups. Formal interaction testing indicated a significant effect modification by hypertension (*p* for interaction = 0.020), with a stronger association observed in non‐hypertensive patients (HR 1.37, 95% CI 1.19−1.59) compared with those with hypertension (HR 1.11, 95% CI 1.00−1.23). A borderline interaction was also observed for smoking status (*p* for interaction = 0.049). No significant interactions were detected for age, sex, BMI, diabetes, dyslipidemia, multivessel CAD, or lesion length ≥ 20 mm (all *p* for interaction ≥ 0.05). These findings should be interpreted with caution given the exploratory nature of subgroup analyses and the potential for multiple comparisons.

**Table 4:** Subgroup analysis of the association between CTI index and the primary endpoint.

	*N* (%)	HR	95% CI	*p* value	*p* for interaction
Age					0.852
< 65	1574 (73.31)	1.20	(1.09−1.32)	**< 0.001**	
≥ 65	573 (26.69)	1.18	(0.99−1.41)	0.064	
Gender					0.412
Female	486 (22.64)	1.29	(1.07−1.56)	**0.008**	
Male	1661 (77.36)	1.18	(1.07−1.30)	**< 0.001**	
BMI					0.127
< 24	538 (25.06)	1.32	(1.11−1.57)	**< 0.001**	
≥ 24	1609 (74.94)	1.14	(1.04−1.26)	**0.008**	
Smoking					**0.049**
No	854 (39.78)	1.34	(1.17−1.54)	**< 0.001**	
Yes	1293 (60.22)	1.13	(1.01−1.25)	**0.026**	
Diabetes					0.462
Yes	821 (38.24)	1.12	(0.99−1.27)	0.067	
No (FBG **> **6.1)	203 (9.46)	0.97	(0.74−1.28)	0.843	
No (FBG ≤ 6.1)	1123 (52.31)	1.19	(1.02−1.37)	**0.024**	
Hypertension					**0.020**
No	743 (34.61)	1.37	(1.19−1.59)	**< 0.001**	
Yes	1404 (65.39)	1.11	(1.00−1.23)	**0.046**	
Dyslipidemia					0.393
No	240 (11.18)	1.05	(0.78−1.41)	0.753	
Yes	1907 (88.82)	1.21	(1.11−1.32)	**< 0.001**	
Multivessel CAD					0.724
No	414 (19.28)	1.13	(0.88−1.46)	0.346	
Yes	1733 (80.72)	1.19	(1.08−1.29)	**< 0.001**	
Lesion length ≥ 20 mm					0.355
No	480 (22.36)	1.29	(1.05−1.58)	**0.014**	
Yes	1667 (77.64)	1.16	(1.06−1.28)	**0.001**	

*Note:* Bold values indicate statistical significance (*P* < 0.05).

Abbreviations: BMI, body mass index; CAD, coronary artery disease; CI, confidence interval; HR, hazard ratio.

### 3.3 Association Between the CTI Index and the Secondary Endpoints

Repeat revascularization was observed in 930 participants, of whom 387 (41.6%) underwent repeat intervention due to ISR, while the remaining 543 (58.4%) underwent revascularization for non‐ISR‐related causes. Kaplan−Meier analysis showed significantly higher incidence of repeat revascularization across increasing CTI tertiles (log‐rank test *p* < 0.001; Figure 2). In Model 1, CTI index demonstrated significant correlation with repeat revascularization when analyzed as a continuous variable. Multivariable adjustment in Models 2 and 3 revealed this predictor to be independently associated with the outcome (*p* < 0.05 for both). Compared with T_1_, patients in T_3_ had a higher hazard of repeat revascularization in Model 2 (HR, 1.38; 95% CI, 1.16–1.64; *p* < 0.001) and Model 3 (HR, 1.35; 95% CI, 1.14–1.61; *p* < 0.001; Supporting Information S1: Table S6). In contrast, TyG also demonstrated significant predictive value. In Model 3, although the T_3_ group of the TyG index remained at a 24% higher risk versus T_1_ (HR, 1.24; 95% CI 1.04−1.48; *p* = 0.015; Supporting Information S1: Table S6). In all models, CRP failed to retain statistical significance. Figure 3B presents the RCS‐fitted curve for CTI index against repeat revascularization risk in Model 3. A linear dose−response pattern emerged, with the overall association reaching significance (*p* = 0.013), while the nonlinearity test indicating no violation of linear assumption (*p* = 0.079).

ISR occurred in 449 participants, of whom 387 (86.2%) subsequently underwent repeat revascularization. Kaplan−Meier curves revealed a graded increase in ISR incidence across CTI tertiles (log‐rank *p* = 0.003; Figure 2). In Cox regression, CTI index demonstrated significant association with ISR in the unadjusted model, retaining independent predictive capacity after multivariable adjustment (Models 2−3, all *p* < 0.05). In the fully adjusted model, patients in the highest CTI tertile had a significantly higher risk of ISR than those in the lowest tertile (HR 1.34, 95% CI 1.05−1.71, *p* = 0.018; Supporitng Information S1: Table S7). The highest TyG tertile showed a numerically higher risk of ISR, although the association did not reach statistical significance (HR 1.27, 95% CI 0.99−1.63; *p* = 0.057). In all models, CRP failed to retain statistical significance. RCS modeling in Model 3 indicated linear dose−response dependency without significant nonlinearity (nonlinear *p* = 0.118, *p* overall = 0.021; Figure 3C). Detailed Cox regression results appear in Supporting Information S1: Tables S6 and S7.

Supporting Information S1: Tables S8 and S9 present the associations between the CTI index and the secondary endpoints across prespecified subgroups. Overall, the direction of the associations was broadly consistent across most subgroups. Formal interaction testing identified significant effect modification by hypertension (*p* for interaction = 0.006) and multivessel CAD (*p* for interaction = 0.027). For repeat revascularization, the association between CTI and risk appeared stronger in patients without hypertension (HR 1.42, 95% CI 1.23−1.64) compared with those with hypertension (HR 1.10, 95% CI 0.99−1.22). For ISR, the association between CTI and risk was more evident among patients with multivessel disease (HR 1.29, 95% CI 1.13−1.47), whereas the estimate was less precise in those without multivessel disease (HR 0.83, 95% CI 0.57−1.21). No significant interactions were observed for sex, age, BMI, diabetes, dyslipidemia, smoking, or lesion length ≥ 20 mm (all *p* for interaction > 0.05). These subgroup findings should be interpreted with caution given the exploratory nature of the analyses and the potential for multiple comparisons.

## 4 Discussion

This study evaluated the association between the combined inflammatory and IR marker—the CTI index—and long‐term PCI‐related outcomes in patients with CCS undergoing DES implantation. During a median follow‐up of up to 60 months, a higher baseline CTI index was independently associated with an increased risk of repeat revascularization and ISR, with evidence of a linear dose–response relationship. In multivariable analyses, patients in the highest CTI tertile had an approximately 31% higher risk of the primary endpoint compared with those in the lowest tertile. These associations remained robust after adjustment for a broad range of clinical, laboratory, and angiographic characteristics, and were further supported by sensitivity analyses. These findings support an independent association between CTI and adverse post‐PCI outcomes. However, its incremental predictive value beyond established clinical risk factors remains limited.

The CTI index integrates two key indicators—IR (via the TyG index) and systemic inflammatory status (via CRP). Biologically, IR and inflammation form a self‐reinforcing loop that jointly drives atherosclerotic plaque vulnerability and derails vascular repair [37, 38]. IR is one of the core driving factors for the occurrence and development of atherosclerosis [11]. Under conditions of IR, impaired insulin signaling reduces nitric oxide bioavailability and promotes endothelial dysfunction, vascular remodeling, inflammation, and fibrosis, thereby accelerating atherosclerotic progression [39, 40]. In addition, IR is closely related to inflammation and often forms a vicious cycle, aggravating metabolic dysfunction [38, 41, 42]. Sustained inflammatory responses lead to adverse changes in cardiac structure and function, including cardiac remodeling, fibrosis, and reduced contractility [43]. The interaction between IR and chronic inflammation promotes oxidative stress and vascular injury, leading to impaired clearance of apoptotic cells, enlargement of the necrotic core, and increased plaque vulnerability [12, 41, 44]. From a clinical perspective, chronic low‐grade inflammation acts as a key accelerator throughout the entire course of atherosclerosis, not only contributing to plaque destabilization and rupture but also directly promoting neointimal hyperplasia, serving as the primary pathological basis for adverse complications after PCI [10, 45]. Therefore, the CTI index may capture the synergistic pathological processes that lead to adverse events after PCI more comprehensively than a single indicator. Our research findings support the view that patients with both high IR and high inflammatory conditions are more prone to dysregulation of the vascular repair process, thereby leading to a higher demand for revascularization.

Notably, both CTI and TyG remained significantly associated with adverse outcomes after multivariable adjustment, whereas CRP alone did not. This finding suggests that IR‐related metabolic abnormalities may contribute more substantially to the observed associations. Nevertheless, IR and systemic inflammation are closely interconnected and jointly contribute to endothelial dysfunction, oxidative stress, and vascular injury [37, 38]. Therefore, combining TyG and CRP remains biologically plausible, although the inclusion of an inflammatory marker does not necessarily improve predictive performance. The limited additional contribution of CRP may partly reflect its nonspecific nature and the use of a single baseline measurement, which may not adequately capture long‐term inflammatory burden. Furthermore, previous research has reported incremental prognostic value of CTI for major adverse cardiovascular events (MACE) after PCI [46], whereas our study focused specifically on repeat revascularization and ISR. Differences in study populations, outcome definitions, and follow‐up duration may partly explain these inconsistent findings. Overall, our results do not establish the superiority of CTI over TyG alone, and further studies are needed to determine whether combining inflammatory and metabolic markers provides meaningful additional prognostic information.

Subgroup analyses suggested potential heterogeneity in the association between the CTI index and clinical outcomes. A significant interaction was observed for hypertension (*p* for interaction = 0.020), with a relatively stronger association in patients without hypertension. A borderline interaction was also noted for smoking status (*p* for interaction = 0.049). These findings should be interpreted with caution, as multiple subgroup comparisons were performed and statistical power may be limited in some strata. One possible explanation is that established cardiovascular risk factors, such as hypertension and smoking, may contribute substantially to overall risk and thereby attenuate the relative contribution of the metabolic‐inflammatory pathway reflected by the CTI index. In contrast, in individuals without these risk factors, the association between CTI and outcomes may appear more pronounced. Nevertheless, these interpretations remain speculative and require confirmation in future studies. No significant interaction was observed for diabetes status (*p* for interaction = 0.462), suggesting that the association between CTI and the primary endpoint was generally consistent in patients with and without diabetes, despite differences in absolute event rates. Similar patterns were observed for secondary endpoints. Hypertension modified the association between CTI and repeat revascularization (*p* for interaction = 0.006), while multivessel CAD showed a significant interaction for ISR (*p* for interaction = 0.027). The association between CTI and ISR appeared more evident in patients with multivessel disease, possibly reflecting a greater interplay between systemic metabolic‐inflammatory status and complex coronary anatomy. Overall, these subgroup findings are exploratory and hypothesis‐generating in nature and should be interpreted cautiously, particularly in light of multiple comparisons and limited statistical power.

The multivariable clinical prediction model constructed in this study demonstrated modest discriminative ability, with AUCs of 0.627 and 0.651 for 3‐ and 5‐year predictions, respectively, and a *C*‐index of 0.609. These findings suggest that although the CTI index was independently associated with the risk of PCI‐related complications, the overall ability of the model to discriminate individual risk remains limited. This observation is not unexpected, given the multifactorial nature of post‐PCI restenosis and repeat revascularization, which are influenced by a complex interplay of anatomical characteristics, procedural factors, lesion complexity, medication adherence, and biological processes that were not fully captured in the present study. To further evaluate the incremental prognostic value of CTI, we compared the baseline clinical model with and without the CTI index. Although the addition of CTI resulted in numerically higher AUCs and *C*‐statistics, the improvements in NRI, IDI, and LRT did not reach statistical significance. These findings indicate that the incremental predictive gain provided by CTI beyond established clinical risk factors may be modest. Nevertheless, decision curve analysis suggested a potential increase in net benefit across a range of clinically relevant threshold probabilities. Therefore, CTI may provide complementary information for risk stratification when used in conjunction with established clinical risk factors. Importantly, CTI is derived from routinely available and inexpensive laboratory parameters that reflect both IR and systemic inflammation, two key mechanisms implicated in atherosclerotic progression and restenosis. Therefore, even modest improvements in predictive performance may be clinically relevant when achieved using a simple and readily accessible biomarker. Taken together, our findings suggest that the principal value of CTI may lie in complementing conventional risk assessment rather than serving as a standalone prognostic marker, thereby providing additional information for individualized risk stratification following PCI.

Compared to previous similar studies [46], the present study has several distinguishing features. First, unlike conventional cardiovascular prognostic studies primarily focused on MACE, we specifically focused on long‐term lesion‐oriented vascular complications—angiographically confirmed ISR and clinically driven repeat revascularization—as the primary endpoint in CCS patients after DES implantation. This endpoint selection was based on the clinical context of PCI‐treated populations, in whom adverse prognosis frequently manifests as recurrent coronary lesion progression and stent failure rather than hard ischemic events alone. Moreover, these outcomes are clinically meaningful manifestations of adverse vascular healing, neointimal hyperplasia, and neoatherosclerosis after PCI, thereby potentially providing a more sensitive assessment of residual coronary risk. Second, methodologically, we not only validated the prognostic value of baseline CTI but also developed and internally validated a multivariable clinical prediction model that integrates CTI with clinical and angiographic characteristics. We further quantified the model's net clinical benefit using DCA, thereby advancing the field from biomarker association toward practical utility assessment. Third, exploratory subgroup analyses suggested potential differences in the association between CTI and clinical outcomes according to hypertension status, although these findings require further validation.

From a clinical perspective, CTI is derived from routinely available laboratory parameters and may have potential value for risk stratification after PCI. This study showed that patients in the highest tertile (CTI ≥ 9.64) had a significantly higher risk of clinical endpoints than those in the lowest tertile. This finding suggests that patients with elevated CTI index may warrant closer surveillance and more comprehensive cardiovascular risk assessment and more intensive management of cardiovascular risk factors. However, because this was an observational study and no formal cutoff analysis was performed, the threshold identified in the present cohort should not be interpreted as a clinically validated cutoff value. Although CTI was independently associated with adverse outcomes, it is unlikely to replace existing risk assessment strategies. However, it may provide complementary information by integrating both inflammatory and metabolic components. Therefore, CTI may offer a broader assessment of cardiometabolic risk than traditional implements. Whether CTI‐guided management can improve clinical outcomes remains uncertain and warrants further investigation in prospective studies.

This study has several limitations. First, as a single‐center retrospective study conducted at a high‐volume specialized cardiac center in China, the present study is subject to inherent selection bias, and the generalizability of the findings to other ethnic populations, healthcare systems, and clinical settings remains uncertain. Differences in patient characteristics, clinical practice patterns, and healthcare delivery may influence the performance of the CTI‐based prediction model. In particular, because follow‐up CAG or CCTA was required for endpoint assessment, the exclusion of patients without imaging follow‐up may have introduced selection and surveillance bias. Patients undergoing imaging may have had more severe symptoms or higher cardiovascular risk, potentially leading to an overestimation of event rates. However, the exclusion of patients with suspected ISR or disease progression who declined further evaluation or intervention may have affected the observed event rates. Importantly, if imaging follow‐up was associated with both baseline CTI‐related characteristics and subsequent outcomes, the estimated HRs could have been either exaggerated or attenuated. Accordingly, both the observed event rates and HR estimates should be interpreted cautiously. Second, although endpoint events were confirmed by imaging, decisions regarding repeat revascularization were partially influenced by clinical judgment and patient preferences, potentially introducing subjectivity. In addition, the use of both CCTA and CAG may have introduced heterogeneity in endpoint ascertainment because of differences in their diagnostic sensitivity and specificity, potentially affecting the detection of ISR. Although suspected ISR on CCTA required confirmation by CAG, patients without significant stenosis on CCTA were classified as event‐free, which may have resulted in outcome misclassification. Moreover, imaging‐detected ISR and clinically indicated repeat revascularization differ in clinical significance, particularly when restenosis is asymptomatic. The lack of detailed information regarding symptom‐driven interventions further limits the interpretation of the composite endpoint. Similarly, detailed information regarding target‐lesion versus non‐target‐lesion revascularization was unavailable; therefore, the relative contributions of restenosis‐related and progression‐related events could not be fully distinguished. Future studies with standardized imaging protocols and more detailed endpoint adjudication are warranted. Third, only baseline CTI values were analyzed, whereas inflammatory and metabolic status may change over time. Longitudinal CTI assessment should be explored in future prospective studies. Likewise, although medication use at discharge was recorded, longitudinal information regarding adherence to lipid‐lowering and antiplatelet therapies was unavailable. Changes in medication use and adherence during follow‐up may have influenced coronary disease progression, potentially introducing residual confounding and affecting the observed associations between CTI and clinical outcomes. Fourth, some potentially relevant variables—including detailed procedural characteristics (e.g., stent type, implantation technique, and intravascular imaging), advanced lipid parameters, and novel inflammatory markers—were unavailable. In addition, substantial baseline differences in diabetes, hypertension, and medication use were observed across CTI tertiles. Although multivariable models adjusted for major clinical and angiographic risk factors, including diabetes and hypertension, residual confounding due to unmeasured factors, differences in disease severity, and treatment patterns cannot be completely excluded. Therefore, the observed associations should be interpreted cautiously and should not be considered evidence of causality. Finally, multiple subgroup and interaction analyses were performed, potentially increasing the risk of false‐positive findings. Accordingly, these exploratory results should be interpreted cautiously and require independent validation. Although internal validation was performed using bootstrap resampling, external validation in an independent cohort was not available.

## 5 Conclusion

In conclusion, among patients with CCS undergoing DES implantation, a higher baseline CTI index was independently associated with an increased risk of repeat revascularization and ISR during long‐term follow‐up. Nevertheless, its incremental predictive value beyond established clinical risk factors remains limited, and its clinical utility requires further validation. Given the observational design and potential residual confounding, these findings should be interpreted with caution and require confirmation in large, multicenter prospective studies.

## Author Contributions

Study concept and design: Wang Jiale, Liu Longcheng, and Ma Lihong. Acquisition, analysis, and interpretation of the data: Wang Jiale. Drafting of the manuscript: Liu Longcheng. Revision: all authors. Each author contributed important intellectual content during the process of manuscript writing. All authors read and approved the final manuscript.

## Ethics Statement

This study was performed in line with the Declaration of Helsinki and was authorized by the Ethics Committee of Fuwai Hospital (Reference Number: 2016‐786). All participants provided written/oral informed consent to participate.

## Consent

All participants provided written/oral informed consent for publication which has been approved by the Ethics Committee of Fuwai Hospital.

## Conflicts of Interest

The authors declare no conflicts of interest.

## Supporting information


Supporting File

## Data Availability

The data sets used and analyzed during the current study are available from the corresponding author upon reasonable request.
