# Supplementary material for: Inflammatory and Insulin Resistance Indicator for Predicting Repeat Revascularization and In‐Stent Restenosis in Patients With Chronic Coronary Syndrome Undergoing Percutaneous Coronary Intervention
Source: Clin Cardiol. 2026 Nov 5;49(11):e70512. doi: 10.1002/clc.70512 (PMC13739133; doi:10.1002/clc.70512)
Supplement: Supplementary file 1 [file CLC-49-e70512-s001.docx]

**Table S1**. Baseline characteristics according to repeat revascularization

| **Variable** | **No such events**  **(n=1217)** | **Repeat revascularization**  **(n=930)** | **P-value** |
| --- | --- | --- | --- |
| Demographics |  |  |  |
| Age (years) | 59.00 (52.00, 65.00) | 58.00 (52.00, 65.00) | 0.080 |
| Male sex, n(%) | 927(76.17) | 734(78.92) | 0.131 |
| BMI (kg/m^2^) | 25.80 (23.83, 27.89) | 25.97 (24.22, 28.10) | 0.026 |
| Risk factors |  |  |  |
| Cigarette smoking, n (%) | 733(60.23) | 560(60.22) | 0.994 |
| Diabetes, n (%) | 422(34.68) | 399(42.90) | <0.001 |
| Hypertension, n (%) | 775(63.68) | 629(67.63) | 0.056 |
| Dyslipidemia, n (%) | 1077(88.50) | 830(89.25) | 0.584 |
| Previous MI, n (%) | 167(13.72) | 186(20) | <0.001 |
| Previous stroke, n (%) | 125(10.27) | 113(12.15) | 0.169 |
| Previous PCI, n (%) | 291(23.91) | 330(35.48) | <0.001 |
| PAD, n (%) | 164(13.48) | 102(10.97) | 0.080 |
| Family history of CAD, n (%) | 220(18.08) | 184(18.78) | 0.316 |
| Clinical presentations |  |  |  |
| Multi-vessel CAD, n (%) | 916(75.27) | 817(87.85) | <0.001 |
| LVEF (%) | 63.00 (60.00, 65.00) | 63.00 (60.00, 65.00) | 0.113 |
| Laboratory measurements |  |  |  |
| WBC(10^9^/L) | 6.40 (5.41, 7.71) | 6.64 (5.70, 7.81) | <0.001 |
| NEUT(10^9^/L) | 3.98 (3.21, 4.95) | 4.22 (3.43, 5.20) | <0.001 |
| LYMPH(10^9^/L) | 1.78 (1.43, 2.22) | 1.78 (1.46, 2.20) | 0.566 |
| MONO(10^9^/L) | 0.38 (0.30, 0.49) | 0.39 (0.31, 0.50) | 0.247 |
| TC (mmol/L) | 3.95 (3.34, 4.61) | 3.95 (3.37, 4.84) | 0.039 |
| LDL-C (mmol/L) | 2.28 (1.79, 2.87) | 2.36 (1.85, 3.05) | 0.009 |
| HDL-C (mmol/L) | 1.08 (0.91, 1.28) | 1.03 (0.89, 1.23) | <0.001 |
| Triglycerides (mmol/L) | 1.44 (1.07, 1.96) | 1.51 (1.14, 2.12) | 0.003 |
| FPG (mmol/L) | 5.52 (5.01, 6.62) | 5.90 (5.11, 7.35) | <0.001 |
| HbA1c (%) | 5.90 (5.60, 6.60) | 6.10 (5.70, 7.10) | <0.001 |
| ESR(mm/h) | 7.00 (3.00, 13.00) | 7.00 (3.00, 14.00) | 0.337 |
| URIC(μmol/L) | 347.91 (292.75, 402.06) | 345.08 (285.33, 399.05) | 0.224 |
| Lp(a)(nmol/L) | 145.49 (66.00, 388.33) | 176.98 (78.33, 459.65) | <0.001 |
| Pcr (μmol/L) | 78.67 (69.02, 89.11) | 79.16 (69.57, 89.81) | 0.239 |
| eGFR (mL/min per 1.73 m^2^) | 108.23 (95.39, 122.06) | 109.65 (93.28, 125.14) | 0.661 |
| NT-proBNP (pg/mL) | 84.20 (34.00, 184.30) | 84.25 (40.02, 201.20) | 0.165 |
| CRP(mg/L) | 2.63 (1.81, 4.56) | 2.81 (1.91, 4.57) | 0.165 |
| hs-CRP (mg/L) | 1.51 (0.79, 3.19) | 1.67 (0.87, 3.34) | 0.035 |
| Medications at discharge |  |  |  |
| DAPT, n (%) | 1211(99.51) | 930(100) | 0.083 |
| Statins, n (%) | 1200(98.60) | 915(98.39) | 0.682 |
| ACEIs, n (%) | 644(52.92) | 501(53.87) | 0.661 |
| β-Blockers, n (%) | 1026(84.31) | 806(86.67) | 0.126 |
| Insulin, n (%) | 104(8.55) | 137(14.73) | <0.001 |
| Oral hypoglycemic drugs, n (%) | 294(24.16) | 261(28.06) | 0.040 |
| Angiographic findings |  |  |  |
| Restenotic lesions, n (%) | 34(2.79) | 50(5.38) | 0.002 |
| Chronic total occlusions, n (%) | 172(14.13) | 171(18.39) | 0.008 |
| Lesions>20 mm long, n (%) | 902(74.12) | 765(82.26) | <0.001 |
| Number of stents | 2.00 (1.00, 2.00) | 2.00 (1.00, 3.00) | <0.001 |
| Length of stent (mm) | 33.00 (19.00, 52.00) | 36.00 (23.00, 58.00) | <0.001 |
| CTI index | 9.24 (8.84, 9.75) | 9.42 (8.97, 9.88) | <0.001 |
| CTI tertiles |  |  | <0.001 |
| T_1_, n (%) | 438(61.17) | 258(36.03) |  |
| T_2_, n (%) | 395(55.24) | 320(44.76) |  |
| T_3_, n (%) | 364(50.84) | 352(49.16) |  |

ACEI, angiotensin-converting enzyme inhibitor; BMI, body mass index; CAD, coronary artery disease; CRP, C-reactive protein; DAPT, dual antiplatelet therapy; ESR, erythrocyte sedimentation rate; eGFR, estimated glomerular filtration rate; FPG, fasting plasma glucose; HDL-C, high-density lipoprotein-cholesterol; HbA1c, glycated hemoglobin A1c; hs-CRP, hypersensitive C-reactive protein; LYMPH, lymphocytes; LVEF, left ventricular ejection fraction; Lp(a), lipoprotein(a); LDL-C, low-density lipoprotein-cholesterol; MONO, monocytes; MI, myocardial infarction; NT-proBNP, N-terminal pro-B-type natriuretic peptide; NEUT, neutrophils; PCI, percutaneous coronary intervention; PAD, peripheral arterial disease; Pcr, plasma creatine; TC, total cholesterol; URIC, uric acid; WBC, white blood cells; CTI, C-Reactive protein-triglyceride glucose index; T, tertile.

**Table S2**. Baseline characteristics according to in-stent restenosis

| **Variable** | **No such events (n=1698)** | **In-stent restenosis(n=449)** | **P-value** |
| --- | --- | --- | --- |
| Demographics |  |  |  |
| Age (years) | 59.00 (52.00, 65.00) | 59.00 (53.00, 65.00) | 0.475 |
| Male sex, n(%) | 1302(76.68) | 359(79.96) | 0.140 |
| BMI (kg/m^2^) | 25.88 (23.88, 28.03) | 25.99 (24.22, 28.04) | 0.201 |
| Risk factors |  |  |  |
| Cigarette smoking, n (%) | 1018(59.95) | 275(61.25) | 0.618 |
| Diabetes, n (%) | 608(35.81) | 213(47.44) | <0.001 |
| Hypertension, n (%) | 1092(64.31) | 312(69.49) | 0.040 |
| Dyslipidemia, n (%) | 1506(88.69) | 401(89.31) | 0.712 |
| Previous MI, n (%) | 242(14.25) | 111(24.72) | <0.001 |
| Previous stroke, n (%) | 191(11.25) | 47(10.47) | 0.639 |
| Previous PCI, n (%) | 399(23.50) | 222(49.44) | <0.001 |
| PAD, n (%) | 200(11.78) | 66(14.70) | 0.095 |
| Family history of CAD, n (%) | 318(18.73) | 86(19.15) | 0.837 |
| Clinical presentations |  |  |  |
| Multi-vessel CAD, n (%) | 1346(79.27) | 387(86.19) | <0.001 |
| LVEF (%) | 63.00 (60.00, 65.00) | 63.00 (60.00, 65.00) | 0.104 |
| Laboratory measurements |  |  |  |
| WBC(10^9^/L) | 6.49 (5.52, 7.73) | 6.65 (5.65, 7.87) | 0.079 |
| NEUT(10^9^/L) | 4.06 (3.28, 5.02) | 4.21 (3.38, 5.27) | 0.074 |
| LYMPH(10^9^/L) | 1.78 (1.45, 2.20) | 1.75 (1.44, 2.22) | 0.598 |
| MONO(10^9^/L) | 0.39 (0.31, 0.49) | 0.39 (0.31, 0.50) | 0.319 |
| TC (mmol/L) | 3.96 (3.35, 4.68) | 3.90 (3.32, 4.79) | 0.693 |
| LDL-C (mmol/L) | 2.32 (1.82, 2.94) | 2.24 (1.78, 2.92) | 0.386 |
| HDL-C (mmol/L) | 1.07 (0.91, 1.27) | 1.03 (0.88, 1.24) | 0.017 |
| Triglycerides (mmol/L) | 1.45 (1.09, 1.98) | 1.53 (1.13, 2.26) | 0.026 |
| FPG (mmol/L) | 5.60 (5.03, 6.79) | 6.01 (5.18, 7.63) | <0.001 |
| HbA1c (%) | 5.90 (5.60, 6.70) | 6.30 (5.70, 7.40) | <0.001 |
| ESR(mm/h) | 7.00 (3.00, 13.00) | 7.00 (3.00, 14.00) | 0.543 |
| URIC(μmol/L) | 347.00 (289.52, 401.31) | 346.23 (291.20, 399.89) | 0.928 |
| Lp(a)(nmol/L) | 152.31 (67.60, 398.94) | 186.13 (76.70, 484.36) | 0.013 |
| Pcr (μmol/L) | 78.81 (69.02, 89.23) | 79.13 (69.60, 90.42) | 0.351 |
| eGFR (mL/min per 1.73 m^2^) | 108.91 (94.87, 122.84) | 108.29 (93.73, 125.10) | 0.922 |
| NT-proBNP (pg/mL) | 80.00 (34.40, 179.23) | 95.80 (44.60, 223.70) | 0.001 |
| CRP(mg/L) | 2.70 (1.85, 4.60) | 2.81 (1.91, 4.45) | 0.874 |
| hs-CRP (mg/L) | 1.57 (0.83, 3.30) | 1.63 (0.82, 3.10) | 0.851 |
| Medications at discharge |  |  |  |
| DAPT, n (%) | 1694(99.76) | 447(99.55) | 0.805 |
| Statins, n (%) | 1677(98.76) | 438(97.55) | 0.059 |
| ACEIs, n (%) | 896(52.77) | 249(55.46) | 0.310 |
| β-Blockers, n (%) | 1448(85.28) | 384(85.52) | 0.896 |
| Insulin, n (%) | 174(10.25) | 67(14.92) | 0.005 |
| Oral hypoglycemic drugs, n (%) | 416(24.50) | 139(30.96) | 0.005 |
| Angiographic findings |  |  |  |
| Restenotic lesions, n (%) | 37(2.18) | 47(10.47) | <0.001 |
| Chronic total occlusions, n (%) | 258(15.19) | 85(18.93) | 0.055 |
| Lesions>20 mm long, n (%) | 1290(75.97) | 377(83.96) | <0.001 |
| Number of stents | 2.00 (1.00, 2.00) | 2.00 (1.00, 3.00) | <0.001 |
| Length of stent (mm) | 33.00 (20.00, 52.00) | 40.00 (25.00, 65.00) | <0.001 |
| CTI index | 9.30 (8.88, 9.78) | 9.42 (8.94, 9.93) | <0.001 |
| CTI tertiles |  |  | 0.002 |
| T_1_, n (%) | 586(81.84) | 130(18.16) |  |
| T_2_, n (%) | 577(80.70) | 138(19.30) |  |
| T_3_, n (%) | 535(74.72) | 181(25.28) |  |

ACEI, angiotensin-converting enzyme inhibitor; BMI, body mass index; CAD, coronary artery disease; CRP, C-reactive protein; DAPT, dual antiplatelet therapy; ESR, erythrocyte sedimentation rate; eGFR, estimated glomerular filtration rate; FPG, fasting plasma glucose; HDL-C, high-density lipoprotein-cholesterol; HbA1c, glycated hemoglobin A1c; hs-CRP, hypersensitive C-reactive protein; LYMPH, lymphocytes; LVEF, left ventricular ejection fraction; Lp(a), lipoprotein(a); LDL-C, low-density lipoprotein-cholesterol; MONO, monocytes; MI, myocardial infarction; NT-proBNP, N-terminal pro-B-type natriuretic peptide; NEUT, neutrophils; PCI, percutaneous coronary intervention; PAD, peripheral arterial disease; Pcr, plasma creatine; TC, total cholesterol; URIC, uric acid; WBC, white blood cells; CTI, C-Reactive protein-triglyceride glucose index; T, tertile.

**Table S3** Associations between the CTI index and endpoints in complete case(n = 2,146)

|  | **Model 3** | | |
| --- | --- | --- | --- |
|  | **HR** | **95%CI** | **P-value** |
| **The primary endpoint** |  |  |  |
| CTI index | 1.11 | 1.01-1.22 | **0.029** |
| CTI tertiles |  |  |  |
| T_1_ | Reference |  |  |
| T_2_ | 1.25 | 1.06-1.47 | **0.007** |
| T_3_ | 1.31 | 1.10-1.55 | **0.002** |
| **Repeat revascularization** |  |  |  |
| CTI index | 1.13 | 1.02-1.24 | **0.015** |
| CTI tertiles |  |  |  |
| T_1_ | Reference |  |  |
| T_2_ | 1.28 | 1.08-1.51 | **0.004** |
| T_3_ | 1.35 | 1.14-1.61 | **<0.001** |
| **ISR** |  |  |  |
| CTI index | 1.16 | 1.01-1.33 | **0.033** |
| CTI tertiles |  |  |  |
| T_1_ | Reference |  |  |
| T_2_ | 1.03 | 0.80-1.31 | **0.854** |
| T_3_ | 1.34 | 1.05-1.71 | **0.020** |

Model 3: adjusted for age, gender, BMI, diabetes, hypertension, previous PCI, previous MI, HbA1c, Lp(a), HDL-C, presence of multivessel CAD, presence of lesion’s length≥20 mm, number of stents, stent length.

Complete-case analysis was performed including only patients with no missing data in any covariates included in the multivariable model. BMI, body mass index; CTI, C-Reactive protein-triglyceride glucose index; CI, confidence interval; CAD, coronary artery disease; HbA1c, glycated hemoglobin A1c; HR, hazard ratio; HDL-C, high-density lipoprotein-cholesterol; Lp(a), lipoprotein(a); MI, myocardial infarction; PCI, percutaneous coronary intervention; ISR, in-stent restenosis.

**Table S4** Sensitivity analysis after excluding patients with prior MI, prior PCI, restenotic lesions, and chronic total occlusions lesions(n = 1,196)

|  | **Model 1** | | | **Model 2** | | | **Model 3** | | |
| --- | --- | --- | --- | --- | --- | --- | --- | --- | --- |
|  | HR | 95%CI | P-value | HR | 95%CI | P-value | HR | 95%CI | P-value |
| **The primary endpoint** |  |  |  |  |  |  |  |  |  |
| CTI index | 1.24 | 1.10-1.41 | **<0.001** | 1.20 | 1.04-1.38 | **0.012** | 1.18 | 1.03-1.36 | **0.018** |
| CTI tertiles |  |  |  |  |  |  |  |  |  |
| T_1_ | Reference |  |  | Reference |  |  | Reference |  |  |
| T_2_ | 1.33 | 1.05-1.68 | **0.017** | 1.29 | 1.02-1.66 | **0.033** | 1.27 | 1.00-1.62 | 0.053 |
| T_3_ | 1.64 | 1.31-2.06 | **<0.001** | 1.55 | 1.21-1.99 | **<0.001** | 1.54 | 1.20-1.98 | **<0.001** |
| **Repeat revascularization** |  |  |  |  |  |  |  |  |  |
| CTI index | 1.25 | 1.10-1.42 | **<0.001** | 1.20 | 1.04-1.38 | **0.013** | 1.18 | 1.03-1.36 | **0.021** |
| CTI tertiles |  |  |  |  |  |  |  |  |  |
| T_1_ | Reference |  |  | Reference |  |  | Reference |  |  |
| T_2_ | 1.36 | 1.07-1.72 | **0.013** | 1.32 | 1.03-1.68 | **0.029** | 1.29 | 1.01-1.65 | **0.044** |
| T_3_ | 1.66 | 1.31-2.09 | **<0.001** | 1.54 | 1.20-1.99 | **<0.001** | 1.53 | 1.19-1.98 | **<0.001** |
| **ISR** |  |  |  |  |  |  |  |  |  |
| CTI index | 1.28 | 1.03-1.58 | **0.024** | 1.33 | 1.05-1.69 | **0.019** | 1.35 | 1.06-1.72 | **0.015** |
| CTI tertiles |  |  |  |  |  |  |  |  |  |
| T_1_ | Reference |  |  | Reference |  |  | Reference |  |  |
| T_2_ | 1.31 | 0.87-1.98 | 0.195 | 1.38 | 0.90-2.11 | 0.136 | 1.32 | 0.87-2.02 | 0.194 |
| T_3_ | 1.76 | 1.19-2.59 | **0.005** | 1.89 | 1.22-2.91 | **0.004** | 1.91 | 1.24-2.95 | **0.003** |

Model 1: unadjusted.

Model 2: adjusted for age, gender, BMI, diabetes, hypertension, HbA1c, Lp(a), HDL-C.

Model 3: adjusted for age, gender, BMI, diabetes, hypertension, HbA1c, Lp(a), HDL-C, presence of multivessel CAD, presence of lesion’s length≥20 mm, number of stents, stent length.

**Among the original cohort of 2,147 patients, 951 high-risk patients (including those with prior MI, prior PCI, restenotic lesions, or chronic total occlusions lesions) were excluded, leaving 1,196 patients for the sensitivity analysis.** BMI, body mass index; CTI, C-Reactive protein-triglyceride glucose index; CI, confidence interval; CAD, coronary artery disease; HbA1c, glycated hemoglobin A1c; HR, hazard ratio; HDL-C, high-density lipoprotein-cholesterol; Lp(a), lipoprotein(a); MI, myocardial infarction; PCI, percutaneous coronary intervention; ISR, in-stent restenosis.

**Table S5. Incremental prognostic performance of CTI added to the baseline model for the primary endpoint**

|  | **3 years** | | **5 years** | | **C-statistic（95%CI）** | **NRI（95%CI）** | P-value | **IDI（95%CI）** | P-value | **LRT** P-value |
| --- | --- | --- | --- | --- | --- | --- | --- | --- | --- | --- |
|  | **AUC** | **95%CI** | **AUC** | **95%CI** |  |  |  |  |  |  |
| **Primary endpoint** |  |  |  |  |  |  |  |  |  |  |
| baseline model | 0.626 | 0.602-0.650 | 0.649 | 0.625-0.672 | 0.608  (0.590-0.625) | - | - | - | - | - |
| baseline model  +CTI | 0.627 | 0.602-0.651 | 0.651 | 0.627-0.674 | 0.609  (0.592-0.626) | 0.054  (-0.021-0.095) | 0.160 | 0.0014  (-0.0001-0.0053) | 0.093 | 0.0716 |

Baseline model included age, gender, BMI, diabetes, hypertension, previous PCI, previous myocardial infarction, HbA1c, lipoprotein(a), HDL-C, presence of multivessel coronary artery disease, lesion length ≥20 mm, number of stents, and total stent length.

BMI, body mass index; CTI, C-Reactive protein-triglyceride glucose index; HbA1c, glycated hemoglobin A1c; HR, hazard ratio; PCI, percutaneous coronary intervention; HDL-C, high-density lipoprotein-cholesterol; AUC, area under the receiver operating characteristic curve; CI, confidence interval; IDI, integrated discrimination improvement; LRT, likelihood-ratio test; NRI, net reclassification improvement.

**Table S6.** Associations between the CTI index and repeat revascularization

|  | Model 1 | | | Model 2 | | | Model 3 | | |
| --- | --- | --- | --- | --- | --- | --- | --- | --- | --- |
|  | HR | 95%CI | P-value | HR | 95%CI | P-value | HR | 95%CI | P-value |
| CTI index | 1.20 | 1.10-1.31 | <0.001 | 1.15 | 1.04-1.26 | 0.005 | 1.13 | 1.02-1.24 | 0.014 |
| CTI tertiles |  |  |  |  |  |  |  |  |  |
| T_1_ | Reference |  |  | Reference |  |  | Reference |  |  |
| T_2_ | 1.32 | 1.12-1.56 | <0.001 | 1.29 | 1.09-1.52 | 0.003 | 1.28 | 1.08-1.51 | 0.004 |
| T_3_ | 1.47 | 1.25-1.72 | <0.001 | 1.38 | 1.16-1.64 | <0.001 | 1.35 | 1.14-1.61 | <0.001 |
| TyG index | 1.27 | 1.15-1.40 | <0.001 | 1.20 | 1.07-1.35 | 0.002 | 1.18 | 1.06-1.33 | 0.004 |
| TyG tertiles |  |  |  |  |  |  |  |  |  |
| T_1_ | Reference |  |  | Reference |  |  | Reference |  |  |
| T_2_ | 1.18 | 1.01-1.39 | 0.045 | 1.14 | 0.96-1.34 | 0.130 | 1.14 | 0.97-1.35 | 0.118 |
| T_3_ | 1.38 | 1.18-1.62 | <0.001 | 1.27 | 1.07-1.51 | 0.007 | 1.24 | 1.04-1.48 | 0.015 |
| CRP | 1.00 | 0.99-1.00 | 0.505 | 1.00 | 0.99-1.00 | 0.462 | 1.00 | 0.99-1.00 | 0.314 |
| CRP tertiles |  |  |  |  |  |  |  |  |  |
| T_1_ | Reference |  |  | Reference |  |  | Reference |  |  |
| T_2_ | 1.01 | 0.86-1.18 | 0.906 | 0.99 | 0.84-1.16 | 0.854 | 0.97 | 0.82-1.14 | 0.679 |
| T_3_ | 1.06 | 0.90-1.24 | 0.492 | 1.03 | 0.88-1.21 | 0.707 | 1.01 | 0.86-1.19 | 0.905 |

Model 1: unadjusted.

Model 2: adjusted for age, gender, BMI, diabetes, hypertension, previous PCI, previous MI, HbA1c, Lp(a), HDL-C.

Model 3: adjusted for age, gender, BMI, diabetes, hypertension, previous PCI, previous MI, HbA1c, Lp(a), HDL-C, presence of multivessel CAD, presence of lesion’s length≥20 mm, number of stents, stent length.

BMI, body mass index; CTI, C-Reactive protein-triglyceride glucose index; CI, confidence interval; CAD, coronary artery disease; HbA1c, glycated hemoglobin A1c; HR, hazard ratio; HDL-C, high-density lipoprotein-cholesterol; Lp(a), lipoprotein(a); MI, myocardial infarction; PCI, percutaneous coronary intervention; TyG, triglyceride-glucose index; CRP, C-reactive protein.

**Table S7.** Associations between the CTI index and in-stent restenosis

|  | Model 1 | | | Model 2 | | | Model 3 | | |
| --- | --- | --- | --- | --- | --- | --- | --- | --- | --- |
|  | HR | 95%CI | P-value | HR | 95%CI | P-value | HR | 95%CI | P-value |
| CTI index | 1.23 | 1.09-1.40 | <0.001 | 1.18 | 1.03-1.35 | 0.020 | 1.17 | 1.02-1.34 | 0.028 |
| CTI tertiles |  |  |  |  |  |  |  |  |  |
| T_1_ | Reference |  |  | Reference |  |  | Reference |  |  |
| T_2_ | 1.05 | 0.83-1.33 | 0.694 | 1.04 | 0.81-1.32 | 0.811 | 1.02 | 0.80-1.31 | 0.856 |
| T_3_ | 1.42 | 1.13-1.78 | 0.002 | 1.35 | 1.06-1.73 | 0.016 | 1.34 | 1.05-1.71 | 0.018 |
| TyG index | 1.36 | 1.17-1.57 | <0.001 | 1.26 | 1.07-1.48 | 0.006 | 1.26 | 1.07-1.48 | 0.006 |
| TyG tertiles |  |  |  |  |  |  |  |  |  |
| T_1_ | Reference |  |  | Reference |  |  | Reference |  |  |
| T_2_ | 1.04 | 0.82-1.32 | 0.768 | 1.01 | 0.79-1.28 | 0.982 | 1.01 | 0.79-1.29 | 0.921 |
| T_3_ | 1.43 | 1.14-1.79 | 0.002 | 1.29 | 1.01-1.65 | 0.043 | 1.27 | 0.99-1.63 | 0.057 |
| CRP | 1.00 | 0.99-1.01 | 0.494 | 1.00 | 0.99-1.01 | 0.687 | 1.00 | 0.99-1.01 | 0.564 |
| CRP tertiles |  |  |  |  |  |  |  |  |  |
| T_1_ | Reference |  |  | Reference |  |  | Reference |  |  |
| T_2_ | 1.05 | 0.83-1.31 | 0.687 | 1.04 | 0.82-1.30 | 0.768 | 1.01 | 0.80-1.27 | 0.944 |
| T_3_ | 1.02 | 0.81-1.28 | 0.857 | 1.07 | 0.85-1.35 | 0.589 | 1.03 | 0.82-1.31 | 0.785 |

Model 1: unadjusted.

Model 2: adjusted for age, gender, BMI, diabetes, hypertension, previous PCI, previous MI, HbA1c, Lp(a), HDL-C.

Model 3: adjusted for age, gender, BMI, diabetes, hypertension, previous PCI, previous MI, HbA1c, Lp(a), HDL-C, presence of multivessel CAD, presence of lesion’s length≥20 mm, number of stents, stent length.

BMI, body mass index; CTI, C-Reactive protein-triglyceride glucose index; CI, confidence interval; CAD, coronary artery disease; HbA1c, glycated hemoglobin A1c; HR, hazard ratio; HDL-C, high-density lipoprotein-cholesterol; Lp(a), lipoprotein(a); MI, myocardial infarction; PCI, percutaneous coronary intervention; TyG, triglyceride-glucose index; CRP, C-reactive protein.

**Table S8.** Subgroup analysis of the association between CTI index and repeat revascularization

| **Variables** | **N (%)** | **HR (95%CI)** | **P** | **P for interaction** |
| --- | --- | --- | --- | --- |
|  |  |  |  |  |
| Gender |  |  |  | 0.827 |
| Female | 486 (22.64) | 1.23 (1.01 ~ 1.50) | 0.039 |  |
| Male | 1661 (77.36) | 1.20 (1.09 ~ 1.32) | <0.001 |  |
| Age |  |  |  | 0.542 |
| ＜65 | 1574 (73.31) | 1.22 (1.10 ~ 1.34) | <0.001 |  |
| ≥65 | 573 (26.69) | 1.14 (0.95 ~ 1.37) | 0.150 |  |
| BMI |  |  |  | 0.134 |
| ＜24 | 538 (25.06) | 1.33 (1.12 ~ 1.58) | 0.001 |  |
| ≥24 | 1609 (74.94) | 1.15 (1.04 ~ 1.27) | 0.008 |  |
| Diabetes |  |  |  | 0.572 |
| Yes | 821 (38.24) | 1.16 (1.02 ~ 1.32) | 0.021 |  |
| No(FBG＞6.1) | 203(9.46) | 0.98 (0.74 ~ 1.30) | 0.908 |  |
| No(FBG≤6.1) | 1123(52.31) | 1.15 (0.99 ~ 1.34) | 0.065 |  |
| Dyslipidemia |  |  |  | 0.360 |
| No | 240 (11.18) | 1.04 (0.77 ~ 1.41) | 0.790 |  |
| Yes | 1907 (88.82) | 1.22 (1.11 ~ 1.33) | <0.001 |  |
| Hypertension |  |  |  | **0.006** |
| No | 743 (34.61) | 1.42 (1.23 ~ 1.64) | <0.001 |  |
| Yes | 1404 (65.39) | 1.10 (0.99 ~ 1.22) | 0.089 |  |
| Smoking |  |  |  | 0.239 |
| No | 854 (39.78) | 1.29 (1.12 ~ 1.49) | <0.001 |  |
| Yes | 1293 (60.22) | 1.16 (1.04 ~ 1.29) | 0.008 |  |
| Multivessel CAD |  |  |  | 0.954 |
| No | 414 (19.28) | 1.19 (0.91 ~ 1.55) | 0.195 |  |
| Yes | 1733 (80.72) | 1.18 (1.08 ~ 1.29) | <0.001 |  |
| Lesion’s length≥20 mm |  |  |  | 0.382 |
| No | 480 (22.36) | 1.29 (1.04 ~ 1.60) | 0.018 |  |
| Yes | 1667 (77.64) | 1.17 (1.06 ~ 1.28) | 0.001 |  |

BMI, body mass index; CAD, coronary artery disease; CI, confidence interval; HR, hazard ratio.

**Table S9.** Subgroup analysis of the association between CTI index and in-stent restenosis

| **Variables** | **n (%)** | **HR (95%CI)** | **P** | **P for interaction** |
| --- | --- | --- | --- | --- |
|  |  |  |  |  |
| Gender |  |  |  | 0.459 |
| Female | 486 (22.64) | 1.12 (0.83 ~ 1.51) | 0.474 |  |
| Male | 1661 (77.36) | 1.26 (1.10 ~ 1.45) | <0.001 |  |
| Age |  |  |  | 0.919 |
| ＜65 | 1574 (73.31) | 1.25 (1.08 ~ 1.44) | 0.003 |  |
| ≥65 | 573 (26.69) | 1.23 (0.95 ~ 1.58) | 0.115 |  |
| BMI |  |  |  | 0.976 |
| ＜24 | 538 (25.06) | 1.21 (0.93 ~ 1.58) | 0.160 |  |
| ≥24 | 1609 (74.94) | 1.22 (1.05 ~ 1.40) | 0.008 |  |
| Diabetes |  |  |  | 0.222 |
| Yes | 821 (38.24) | 1.22 (1.02 ~ 1.46) | 0.026 |  |
| No(FBG＞6.1) | 203(9.46) | 0.80 (0.51 ~ 1.25) | 0.325 |  |
| No(FBG≤6.1) | 1123(52.31) | 1.13 (0.89 ~ 1.42) | 0.313 |  |
| Dyslipidemia |  |  |  | 0.353 |
| No | 240 (11.18) | 1.02 (0.66 ~ 1.58) | 0.921 |  |
| Yes | 1907 (88.82) | 1.25 (1.10 ~ 1.43) | <0.001 |  |
| Hypertension |  |  |  | 0.323 |
| No | 743 (34.61) | 1.34 (1.08 ~ 1.66) | 0.008 |  |
| Yes | 1404 (65.39) | 1.17 (1.01 ~ 1.37) | 0.043 |  |
| Smoking |  |  |  | 0.572 |
| No | 854 (39.78) | 1.29 (1.05 ~ 1.59) | 0.016 |  |
| Yes | 1293 (60.22) | 1.20 (1.03 ~ 1.40) | 0.022 |  |
| Multivessel CAD |  |  |  | **0.027** |
| No | 414 (19.28) | 0.83 (0.57 ~ 1.21) | 0.329 |  |
| Yes | 1733 (80.72) | 1.29 (1.13 ~ 1.47) | <0.001 |  |
| Lesion’s length≥20 mm |  |  |  | 0.887 |
| No | 480 (22.36) | 1.18 (0.86 ~ 1.64) | 0.305 |  |
| Yes | 1667 (77.64) | 1.22 (1.07 ~ 1.40) | 0.004 |  |

BMI, body mass index; CAD, coronary artery disease; CI, confidence interval; HR, hazard ratio.
